# Sodium Selenate Treatment Using a Combination of Seed Priming and Foliar Spray Alleviates Salinity Stress in Rice

**DOI:** 10.3389/fpls.2019.00116

**Published:** 2019-02-11

**Authors:** Kondeti Subramanyam, Gijs Du Laing, Els J. M. Van Damme

**Affiliations:** ^1^Laboratory of Biochemistry and Glycobiology, Department of Biotechnology, Ghent University, Ghent, Belgium; ^2^Laboratory of Analytical Chemistry and Applied Ecochemistry, Department of Green Chemistry and Technology, Ghent University, Ghent, Belgium

**Keywords:** antioxidant enzymes, foliar spray, rice, salinity stress, seed priming, sodium selenate

## Abstract

Soil salinity is one of the important abiotic stress factors that affect rice productivity and quality. Research with several dicotyledonous plants indicated that the detrimental effects associated with salinity stress can (partly) be overcome by the external application of antioxidative substances. For instance, sodium selenate (Na_2_SeO_4_) significantly improved the growth and productivity of several crops under various abiotic stress conditions. At present there is no report describing the impact of Na_2_SeO_4_ on salinity stressed cereals such as rice. Rice cultivation is threatened by increasing salinity stress, and in future this problem will further be aggravated by global warming and sea level rise, impacting coastal areas. The current study reports on the effect of Na_2_SeO_4_ in alleviating salinity stress in rice plants. The optimal concentration of Na_2_SeO_4_ and the most efficient mode of selenium application were investigated. Selenium, sodium, and potassium contents in leaves were determined. Antioxidant enzyme activities as well as proline, hydrogen peroxide (H_2_O_2_), and malondialdehyde (MDA) concentrations were analyzed. In addition, the transcript levels for *OsNHX*1, an important Na^+^/H^+^ antiporter, were quantified. Treatment of 2-week-old rice plants under 150 mM NaCl stress with 6 mg l^-1^ Na_2_SeO_4_ improved the total biomass. A significantly higher biomass was observed for the plants that received Na_2_SeO_4_ by a combination of seed priming and foliar spray compared to the individual treatments. The Na_2_SeO_4_ application enhanced the activity of antioxidant enzymes (SOD, APX, CAT, and GSH-Px), increased the proline content, and reduced H_2_O_2_ and MDA concentrations in plants under NaCl stress. These biochemical changes were accompanied by increased transcript levels for *OsNHX*1 resulting in a higher K^+^/Na^+^ ratio in the rice plants under NaCl stress. The results suggest that Na_2_SeO_4_ treatment alleviates the adverse effect of salinity on rice plant growth through enhancing the antioxidant defense system and increase of *OsNHX*1 transcript levels.

## Introduction

Global agricultural productivity is strongly influenced by several abiotic stress factors including cold, drought, soil salinity, flood, and high temperature. Among these abiotic stresses, soil salinity is one of the most devastating environmental stresses, which causes reduction in the cultivable land, crop productivity and quality (Shrivastava and Kumar, 2015). It has been estimated that 20% of total cultivated and 33% of irrigated agricultural lands worldwide are already affected by high salinity. Global warming and sea level rise will aggravate the problem. Consequently, the salinized areas are gradually increasing every year and are expected to reach 50% by the end of year 2050 (Arun et al., 2016). Salinity affects plant growth and development by imposing osmotic stress on plants, and causes an imbalance in cellular ionic flux resulting from altered Na^+^/K^+^ ratios and Na^+^ and Cl^-^ ion concentrations inside cells (Subramanyam et al., 2011). The ionic influx causes oxidative stress which in turn affects the activity of major cytosolic enzymes by disturbing intracellular potassium homeostasis, and negatively affects photosynthesis (Subramanyam et al., 2012).

Being sessile, plants have developed specific mechanisms that allow them to detect precise environmental changes and respond to complex stress conditions, minimizing damage while conserving valuable resources for growth and reproduction. Perception of salinity stress triggers a variety of plant responses, including the activation of enzymatic and non-enzymatic antioxidant systems (Subramanyam et al., 2012) to mitigate ROS accumulation and oxidative stress. Hence, improving the antioxidant potential of plants is a promising way to combat the undesirable effects of ROS induced oxidative damage (Kasote et al., 2015). Even though genetic engineering and plant transformation are considered as efficient tools in developing salinity stress resistant crops by overexpressing various stress resistance genes, several restrictions, and disputes against the cultivation and consumption of genetically modified crops prompted researchers to develop alternative strategies to overcome the detrimental effects of salinity stress. It has been proven that the external application of several antioxidative substances such as polyamines, coumarin, and glycine betaine plays a significant role in alleviating the salinity induced oxidative stress without manipulating the plant genome (Hasanuzzaman et al., 2014; Saleh and Madany, 2015; Arun et al., 2016).

Selenium (Se) is one among several essential micro nutrients needed for humans and animals, and it is an integral part of the enzyme glutathione peroxidase, that prevents ROS induced oxidative damage (Lobanov et al., 2008). Although, higher plants do not require Se for their growth and development (Valkama et al., 2003; Sors et al., 2005; Yao et al., 2009), supplementation of Se at lower concentrations not only protects plants from ROS induced oxidative damage by activating the antioxidative mechanisms (Iqbal et al., 2015) but also improves the Se content in the edible parts of the plants (Broadley et al., 2010; Pezzarossa et al., 2012). Exogenous Se supplementation significantly improved the growth and development of several crops under various stress conditions by increasing the synthesis of osmoprotectants (Hawrylak-Nowak, 2009), activating the antioxidative and detoxification mechanisms (Hasanuzzaman et al., 2011), and reducing the levels of malondialdehyde (MDA) and hydrogen peroxide (H_2_O_2_) (Iqbal et al., 2015). For instance, low concentrations of Se protected cucumber, rapeseed, canola, and parsley from sodium chloride stress (Hawrylak-Nowak, 2009; Hasanuzzaman et al., 2011; Hashem et al., 2013; Habibi, 2017), rapeseed seedlings, olive, and wheat from drought (Hasanuzzaman and Fujita, 2011; Proietti et al., 2013; Nawaz et al., 2015), pumpkin from UV-B radiation (Germ et al., 2005), sorghum and wheat from high temperature stress (Djanaguiraman et al., 2010; Iqbal et al., 2015). In addition, Se also protected several crops from heavy metal stress by influencing the antioxidant defense mechanisms (Filek et al., 2008; Malik et al., 2012; Feng et al., 2013).

Apart from protecting the plants from several abiotic stresses, the optimum concentration of Se improved plant performance in terms of growth, quality, and yield when plants were grown under optimal growth conditions. The growth promoting response to Se was reported in lettuce, ryegrass, and soybean (Hasanuzzaman et al., 2011). Se promotes the growth of aging seedlings and delays senescence (Hartikainen and Xue, 1999; Xue et al., 2001). Pezzarossa et al. (2012) reported that Se treatment enhanced the fruit development and ripening in peach. Higher yield and better storage quality of tubers was observed in potato plants treated with Se compared to control plants, and this could be related to its antioxidative effect in delaying senescence (Turakainen, 2007). Alhough the Se ions stimulate the growth and protect the plants from several environmental stresses, at a higher concentration (>10 mg K g^-1^ soil) Se acts as a pro-oxidant inducing oxidative stress (Hartikainen et al., 2000). The optimum sodium selenate (Na_2_SeO_4_) concentration depends on the plant species and the type of abiotic stress that the plant is confronted with. In addition, the Se application at different growth stages may also protect the plants exposed to continuous abiotic stresses. Hence, the selection of an optimum concentration and application method for Se are crucial to protect plants from environmental stresses.

Rice (*Oryza sativa* L.) is one of the world’s most important cereal food crops belonging to the Poaceae family and serves as a primary food crop, providing one fifth of the calories to more than 3.5 billion people worldwide (Khush, 2013). Due to the ever increasing population, the global rice demand is increasing and it is estimated to reach 852 million tons in 2035 (Khush, 2013). To meet this demand it is necessary to increase the yield potential of rice per hectare of cultivable land. However, repeated usage of soil for rice cultivation, continuous irrigation, poor water quality, and excessive fertilization, global warming, and sea level rise lead to soil salinization. It may be noted that rice is categorized as a salt-sensitive species (Chinnusamy et al., 2005) and salt stress significantly reduces the yield and exhibits symptoms like leaf chlorosis, premature senescence (Thitisaksakul et al., 2015), delayed flowering and panicle initiation (Grattan et al., 2002), and affects tillers, spikelet number, and grain weight (Khatun and Flowers, 1995). Due to soil salinization, in the near future, rice production may not be sufficient to meet the higher demand. Increasing the cultivation of rice on newer fertile lands is not a valuable option because this may affect the production of other vital crops. Hence, it is worthwhile to improve the salinity tolerance of rice to increase the yield and quality under salinity stress conditions.

Although there are reports available dealing with the role of Na_2_SeO_4_ in abating salinity stress in dicotyledonous plants (Hawrylak-Nowak, 2009; Hasanuzzaman et al., 2011; Hashem et al., 2013; Habibi, 2017), only one report described the role of Na_2_SeO_4_ in alleviating salinity stress in a monocotyledonous plant, i.e., garlic (Astaneh et al., 2017). In this report Na_2_SeO_4_ was applied to the plants either by seed priming, fertigation, or foliar spray. Even though seed priming with Na_2_SeO_4_ can stimulate the stress responses and protect the plantlets from initial stress exposure, additional Na_2_SeO_4_ treatments during the early growth stage either by fertigation or foliar spray may protect the plants from long term exposure to the stress conditions. At present, there are no reports on the role of Na_2_SeO_4_ in alleviating salinity stress for cereal crops. Since the effectiveness of Na_2_SeO_4_ in alleviating the abiotic stresses depends on the plant species and the mode of Se absorption, data cannot be extrapolated between different plants and crops. Given their importance for agriculture, the effect of Na_2_SeO_4_ supply on salinity stress should be confirmed for cereal crops.

The present investigation was carried out to elucidate the role of Na_2_SeO_4_ in counteracting the effects of salinity stress in rice. We compared different methods of exogenous Se supply, in particular foliar spray, seed priming, and the combination of both seed priming and foliar spray to alleviate the salinity stress in rice. The effects of Na_2_SeO_4_ on growth parameters, concentration of proline, H_2_O_2_ and MDA, and the antioxidant enzyme activities in rice tissues were studied.

## Materials and Methods

### Seed Source and Seed Priming

Mature and dried seeds of rice (*O. sativa* L. spp. Japonica cv. Nipponbare; GSOR-100) were obtained from the United States Department of Agriculture-Agricultural Research Service (USDA–ARS), Dale Bumpers National Rice Research Center, Stuttgart, AR, United States. The seeds were dehusked and healthy seeds were handpicked for surface sterilization with 70% ethanol for 5 min, 5% sodium hypochlorite for 30 min. Finally, the seeds were rinsed several times with sterile distilled water. The surface sterilized seeds were incubated in sterile distilled water for 6 h on an orbital shaker with 150 rpm at 28°C, and air dried on sterile filter paper for about 24 h at 30°C. These seeds were designated as “unprimed seeds.”

### Seed Germination and Stress Treatment

The unprimed seeds were incubated on sterile filter paper wetted with sterile distilled water in a plant growth chamber at 30°C and 85% relative humidity for 4 days under complete darkness. The germinated seedlings were sown in PVC tubes (15 cm × 2.5 cm) containing 100 g sand and polymer mixture [(density: 2.65 kg/dm^3^; hardness: 7 Mohs; pH: 7; SiO_2_: 99.5%; Fe_2_O_3_: 0.04%; Al_2_O_3_: 0.20; TiO_2_: 0.03%; K_2_O: 0.03%; and CaO: 0.01%) Sibelco, Antwerp, Belgium], and allowed to grow further in the plant growth room at 28°C under 16 h photoperiod (21 μmol m^-2^ s^-2^) with 85% relative humidity. All seedlings were irrigated 3 times a week for 2 weeks with Hoagland solution (10 mL into each tube). Two-week-old plants were used for the stress experiments. Several groups of plants were foliar sprayed with 100 mL of Na_2_SeO_4_ at different concentrations (2, 4, 6, 8, 10, and 12 mg l^-1^) and covered with polythene paper for 2 days to maintain high humidity. In a similar way, plants were foliar sprayed with 100 mL distilled water and used as control plants. Salinity stress was imposed on the Na_2_SeO_4_ treated and untreated plants by irrigating them 3 times a week for 2 weeks with Hoagland solution containing 150 mM NaCl (10 mL into each tube). Na_2_SeO_4_ untreated plants irrigated with only Hoagland solution (10 mL into each tube) were considered as control plants (Supplementary File S1). The concentration of Na_2_SeO_4_ that yielded plants with a higher biomass was considered as the optimum concentration, and was used in later experiments.

### Different Modes of Exogenous Na_2_SeO_4_ Application to Alleviate NaCl Stress

To determine which mode of exogenous Na_2_SeO_4_ application was most efficient in alleviating NaCl stress we compared foliar spray (mode I), seed priming (mode II), and a combination of seed priming and foliar spray (mode III) (Supplementary Files S2–S4). For mode I, surface sterilized seeds were incubated in 50 mL of sterile distilled water for 6 h on an orbital shaker with 150 rpm at 28°C, and then air dried on sterile filter paper for about 24 h at 30°C. Two-week-old plants were foliar sprayed with 100 mL of 6 mg l^-1^ Na_2_SeO_4_ solution, and covered with polythene paper for 2 days to maintain high humidity. In a similar way, the plants were foliar sprayed with 100 mL distilled water and used as control plants. Salinity stress was imposed for 2 weeks as described above.

For mode II, surface sterilized seeds were incubated in 50 mL of 6 mg l^-1^ of Na_2_SeO_4_ solution (referred as “Se primed”) for 6 h on an orbital shaker with 150 rpm at 28°C, and then air dried on sterile filter paper for about 24 h at 30°C. Two-week-old plants raised from the Se primed and unprimed seeds were subjected to salt stress for 2 weeks as described above.

For mode III, 2-week-old plants raised from the 6 mg l^-1^ Na_2_SeO_4_ primed seeds were sprayed with 100 mL of 6 mg l^-1^ Na_2_SeO_4_ solution, covered with polythene paper for 2 days to maintain high humidity. In a similar way, plants were foliar sprayed with 100 mL distilled water and used as control plants. The control and Na_2_SeO_4_ treated plants were subjected to salinity stress for 2 weeks as described above.

At the end of each experiment, all plants were harvested and the length and fresh weight (FW) of roots and shoots determined. The plant material was immersed in liquid nitrogen and stored frozen at –80°C for elemental, biochemical, and qPCR analysis.

### Determination of Selenium (Se), Sodium (Na), and Potassium (K) Content

The plant material was washed thoroughly with distilled water, dried on filter paper, and then oven dried at 60°C for 48 h. Dried plant material was ground to a fine powder using mortar and pestle and about 0.1–0.4 g of was transferred into a digestion tube containing 10 mL 69% HNO_3_. The digestion tubes were closed and placed in a microwave (MARS 5 CEM) for 25 min in total (1200 W, 600 psi, 195°C for 15 min). After digestion, the extract was filtered through a 0.22 μm filter membrane. The Se content in the digested samples was determined by inductively coupled plasma–MS (ICP-MS, PerkinElmer DRC-e, Sunnyvale, CA, United States). The ICP-MS was fitted with a Babington nebulizer and a cyclonic spray chamber. The optimized instrumental parameters consisted of 1250 W power, plasma argon gas flow with a flow rate of 15 L min^-1^, methane as reaction gas with a flow rate of 0.9 mL min^-1^. Se isotope ^80^Se was used as a control to determine the concentration of total Se in the plant material. The Na and K contents in the digests were measured using an inductively coupled plasma optical emission spectrophotometer (ICP-OES, Varian VISTA-MPX).

### Enzymatic Antioxidant Assays

Shoots collected from 4-week-old plants were used for all the biochemical analysis. Frozen plant material (0.5 g) was ground to a fine powder in liquid nitrogen and homogenized in 1 mL extraction buffer. The homogenate was centrifuged at 13,000 rpm for 20 min at 4°C and the supernatant was collected for enzyme assays. The extraction buffer for superoxide dismutase (SOD) and ascorbate peroxidase (APX) activities was composed of 50 mM sodium phosphate buffer (pH 7.0), 0.1 mM EDTA, 5 mM β-mercaptoethanol, 2% PVP, 5 mM ascorbic acid, and 1 mM PMSF, while the extraction buffer for glutathione reductase (GR), glutathione peroxidase (GSH-Px), and guaiacol peroxidase (GPOX) consisted of 100 mM potassium phosphate buffer (pH 7.0), 0.1 mM EDTA, 2% PVP-40, and 1 mM PMSF. The extraction buffer for the catalase (CAT) assay was the same as for SOD but did not contain 5 mM ascorbic acid. The protein content of the crude extracts was quantified by the protein dye binding assay using bovine serum albumin as the standard (Bradford, 1976).

SOD (EC: 1.15.1.1) activity was measured following the method of Prochazkova et al. (2001). The final reaction contained 50 mM sodium phosphate buffer (pH 7.8), 13 mM methionine, 75 μM NBT, 0.1 mM EDTA, 0.1 mL plant extract and 33 μM riboflavin. SOD activity was measured by monitoring the photo reduction of nitroblue tetrazolium at 560 nm. One unit of enzyme was determined as the amount of enzyme reducing 50% of the absorbance reading compared with the non-enzymatic reaction mixture. The enzyme activity is expressed as Unit mg^-1^ protein min^-1^.

APX (EC: 1.11.1.11) activity was determined following the method described by Nakano and Asada (1981). The final reaction solution contained 50 mM sodium phosphate buffer (pH 7.0), 0.5 mM ascorbic acid, 0.1 mM EDTA, 0.1 mL plant extract, and 2 mM H_2_O_2_. APX activity was measured by observing the decrease in absorbance at 290 nm for 1 min using the extinction coefficient of 2.8 mM^-1^cm^-1^ and the enzyme activity was expressed as nmol ascorbate oxidized mg^-1^ protein min^-1^.

CAT (EC: 1.11.1.6) activity was measured according to the method of Velikova et al. (2000) by monitoring the decrease of absorbance at 240 nm for 1 min. The reaction mixture contained 10 mM potassium phosphate buffer (pH 7.0), 0.1 mL plant extract, and 0.04% H_2_O_2_. The catalase activity was calculated using the extinction coefficient of 39.4 M^-1^cm^-1^ and the activity was expressed as nmol H_2_O_2_ reduced mg^-1^ protein min^-1^.

GR (EC: 1.6.4.2) activity was determined as described by Foyer and Halliwell (1976). The oxidized glutathione (GSSG)-dependent oxidation of NADPH was followed at 340 nm in a reaction mixture containing 100 mM potassium phosphate buffer (pH 7.8), 1 μM EDTA, 0.05 mM NADPH, 50 μl plant extract, and 3 mM GSSG. The GR activity was calculated using the extinction coefficient of 6.2 mM^-1^cm^-1^ and the activity was expressed as nmol NADPH oxidized mg^-1^ protein min^-1^.

GSH-Px (EC: 1.11.1.9) activity was determined as described by Elia et al. (2003) with the reaction mixture containing 100 mM Na-phosphate buffer (pH 7.5), 1 mM EDTA, 1 mM NaN_3_, 0.12 mM NADPH, 2 mM GSH, 1.0 U GR, 0.6 mM H_2_O_2_ and 0.1 mL of plant extract. The oxidation of NADPH was recorded at 340 nm for 1 min and the activity was calculated using the extinction coefficient of 6.62 mM^-1^ cm^-1^ and the activity was expressed as Units min^-1^mg^-1^ protein.

GPOX (EC: 1.11.1.7) activity was determined following the method of Velikova et al. (2000). The final reaction solution contained 10 mM potassium phosphate buffer (pH 7.0), 0.6 mL of 1% aqueous solution of guaiacol, 40 μl plant extract, and 5 mM H_2_O_2_. GPOX activity was calculated following the oxidation of guaiacol at 470 nm using an extinction coefficient of 26.6 mM^-1^ cm^-1^. Enzyme activity was expressed as nmol GDPH product formed mg^-1^ protein min^-1^.

### Estimation of Proline

Free proline content was determined according to the method described by Bates et al. (1973). About 0.5 g of plant material was made into a fine powder in liquid nitrogen and then homogenized in 2 mL of 3% aqueous sulfosalicylic acid. After centrifugation 0.3 mL of supernatant was mixed with 0.3 mL glacial acetic acid and 0.3 mL of acid ninhydrin solution, and then boiled in water bath for 1 h. After cooling in an ice bath, the mixture was mixed with 0.6 mL toluene and vortexed for 1 min. Thereafter, the chromophore-containing toluene was separated from the aqueous phase, and its absorbance was measured at 520 nm against toluene. The proline content was determined from a standard curve of proline and calculated as μmol g^-1^ FW.

### Estimation of Total Chlorophyll Content

Total chlorophyll content was estimated spectrophotometrically according to the method described by Arnon (1949). Fresh leaves (0.2 g) were ground to a fine powder in liquid nitrogen and suspended in 10 mL of 80% acetone. The filtrate was collected and the optical density measured at 645 nm and 663 nm using 80% acetone as a blank. Total chlorophyll content was calculated using the following formula and the results were expressed mg g^-1^ of FW.

$$\begin{array}{c} {Chlorophyll a (mg g}^{-}{}^{1}): [(12{.7 \times A}_{663}) – (2{.6 \times A}_{645})] \times mL acetone/mg leaf tissue\\ {Chlorophyll b (mg g}^{-}{}^{1}): [(22{.9 \times A}_{645}) – (4{.68 \times A}_{663})] \times mL acetone/mg leaf tissue\\ {Total chlorophyll (mg g}^{-}{}^{1}): Chlorophyll a + Chlorophyll b \end{array}$$

### Estimation of Relative Water Content (RWC)

The RWC in the leaves was determined following the method of Weatherley (1950). The uniform sized leaf pieces were washed in distilled water, blotted dry, and FWs were determined. Then the leaf pieces were immersed in distilled water for 4 h. After 4 h the leaves were blotted dry and the turgid weight (TW) was taken. The leaves were kept at 60°C in a hot air oven for 36 h and dry weights (DW) were recorded. The RWC was calculated from the following formula.

$$\begin{array}{c} \mathrm{RWC}(\%) = (FW-DW)/(TW-DW)X100 \end{array}$$

### Estimation of H_2_O_2_ and MDA Concentration

H_2_O_2_ levels were determined as described by Velikova et al. (2000). Crushed leaf material (0.2 g) was homogenized in 800 μl of 0.1% (w/v) trichloroacetic acid (TCA) while incubating on ice. The reaction was carried out in a mixture consisting of 60 μl plant extract, 60 μl 10 mM potassium phosphate buffer (pH 7.0), and 60 μl 1M potassium iodide. The absorbance of the reaction was measured at 390 nm and the H_2_O_2_ concentration was calculated using the standard curve. Results were expressed as μmol g^-1^ of FW.

The level of lipid peroxidation in the leaf tissues was measured by estimating MDA concentration according to the method of Heath and Packer (1968). Fresh leaf samples (0.5 g) were ground to a fine powder and then suspended in 10% (w/v) TCA and centrifuged at 13000 rpm for 12 min. The supernatant was mixed with 10% TCA containing 0.5% thiobarbituric acid (TBA), incubated at 95°C for 30 min, and then cooled for 2 min in an ice bath and centrifuged at 13000 rpm for 10 min. MDA content was calculated by the difference in absorbance at 532 and 600 nm using an extinction coefficient of 155 mM^-1^ cm^-1^ and the results were expressed as nmol g^-1^ of FW.

### Quantitative Real Time-PCR (qRT-PCR)

Total RNA was isolated from the frozen plant material (shoots from 4-week-old plants) using NucleoSpin^®^ RNA purification kit (Macherey-Nagel, Düren, Germany) following the manufacturer’s instructions. To eliminate the residual genomic DNA present in the samples, the total RNA was subjected to DNase I treatment (ThermoScientific, Erembodegem, Belgium). First strand cDNA was synthesized form 2 μg total RNA using MMLV reverse transcriptase (Invitrogen, Carlsbad, CA, United States). The cDNA was diluted 2.5 times and the cDNA quality was checked by reverse transcription–PCR (RT-PCR) using a pair of reference gene primers (Supplementary File S5).

The expression of *OsNHX*1 gene was assessed by quantitative real time PCR (qRT-PCR) with the 96-well CFX Connect^TM^ Real-Time PCR Detection System (Bio-Rad, Hercules, CA, United States) using the SYBR^®^ Green Master mix (Bio-Rad, Veenendaal, Netherlands) and gene specific forward and reverse primers (Supplementary File S5). The qRT-PCR thermal profile consisted of 10 min at 95°C, 45 cycles of 15 s at 95°C, 25 s at 60°C, and 20 s at 72°C, and a melting curve was generated after every qRT-PCR run. *OsEXP*Narsai (LOC_Os07g02340.1), *OsEXP* (LOC_Os03g27010), and *OsEIF5C* (LOC_Os11g21990,1) were used as an internal control to normalize the expression data for the *OsNHX1* gene. Reference gene stability and quality control of the samples were validated in the qBASE^PLUS^ software (Hellemans et al., 2007). For each sample, three biological replicates were analyzed using the mean values of two technical replicates. The results were statistically evaluated with the REST-384 software using the pair wise fixed reallocation randomization test (with 2000 randomizations) (Pfaffl et al., 2002).

### Statistical Analysis

Twenty plants were used for each treatment and each treatment was repeated thrice. The root and shoot length, weight, total biomass, and the biochemical analysis data were analyzed using one-way ANOVA, and the differences contrasted using Duncan’s multiple range tests (DMRT). All statistical analyses were performed at the level of P value less than 0.05 using SPSS 10.0 (SPSS Inc., United States).

## Results

### Selection of Optimal Concentration of Na_2_SeO_4_

**FIGURE 1:**
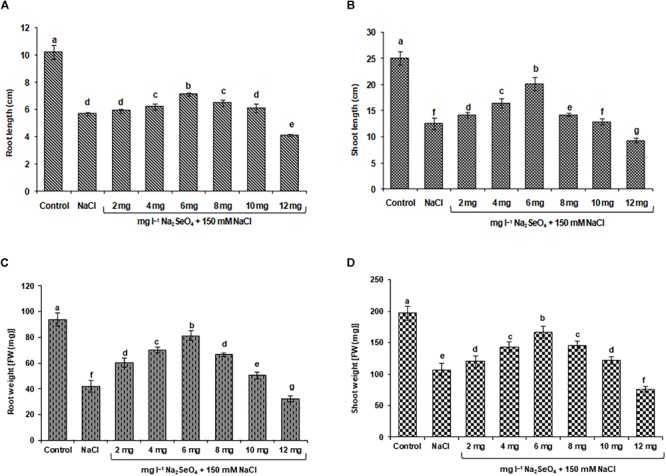
Effect of NaCl stress on the growth of rice plants treated with sodium selenate (Na_2_SeO_4_) by foliar spray. Plants were sprayed with different concentrations of Na_2_SeO_4_ and then exposed to 150 mM NaCl stress for 2 weeks. **(A)** root length (cm), **(B)** shoot length (cm), **(C)** root weight (mg), and **(D)** shoot weight (mg) were quantified. Bars represent the mean values of three independent experiments with standard errors. Means followed by different letters are significantly different according to Duncan’s multiple range test (DMRT) at 5% level.

**FIGURE 2:**
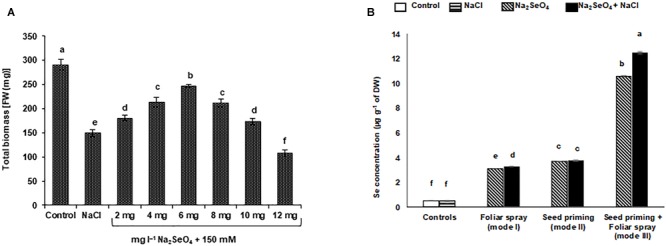
Effect of NaCl stress on the growth response and selenium ion (Se) accumulation of rice plants treated with Na_2_SeO_4_. **(A)** Effect of NaCl stress on total biomass of rice plants treated with Na_2_SeO_4_ by foliar spray. Plants were sprayed with different concentrations of Na_2_SeO_4_ and then exposed to 150 mM NaCl stress for 2 weeks. **(B)** Effect of different modes of Na_2_SeO_4_ fortification on the accumulation of Se (μg g^-1^ DW) in the shoots of rice plants under 150 mM NaCl stress. Bars represent the mean values of three independent experiments with standard errors. Means followed by different letters are significantly different according to Duncan’s multiple range test (DMRT) at 5% level.

After 2 weeks the unstressed rice plants were healthy and were growing well. Conversely, the NaCl treated rice plants (no Na_2_SeO_4_ application) showed toxicity symptoms like stunned growth, chlorosis, necrosis, and leaf burning. Furthermore, a significant reduction in the length (44.2 and 50.2%) and FW (55.4 and 45.6%) of roots and shoots (Figure 1A–D), and total biomass (48.7%) (Figure 2A) was observed in rice plants exposed to the salt stress.

Sodium selenate treatment significantly improved rice performance under NaCl stress. The length and FW of roots and shoots and the total biomass gradually increased with increasing Na_2_SeO_4_ concentration up to 6 mg l^-1^. A concentration of 6 mg l^-1^ Na_2_SeO_4_ was found to be optimal since the length and FW of roots and shoots (Figure 1A–D) and total biomass (Figure 2A) of the rice plants under NaCl stress was higher than for other concentrations tested. Plant treatment with Na_2_SeO_4_ concentrations beyond 6 mg l^-1^ resulted in a gradual reduction of length and FW of roots and shoots, and of total biomass.

### Influence of Mode of Na_2_SeO_4_ Application in the Accumulation of Selenium, Sodium, and Potassium Ions

The average Se content in the non-treated control plants was 0.45 μg g^-1^ of dry weight (DW), which was similar to that of NaCl treated (in absence of Na_2_SeO_4_) plants (Figure 2B). The Na_2_SeO_4_ treatment significantly increased the Se content in both NaCl stressed and unstressed plants. The mode of exogenous Na_2_SeO_4_ supply influenced the Se content in the plants (Figure 2B and Supplementary Table S1). A significantly higher Se content was observed in the plants that received Na_2_SeO_4_ using the combination of seed priming and spraying (mode III; 18.4%) followed by seed priming (mode II; 3.3%) and foliar spray (mode I; 2.1%) compared to plants treated with Na_2_SeO_4_ in the absence of salinity stress (Figure 2B and Supplementary Table S1).

**FIGURE 3:**
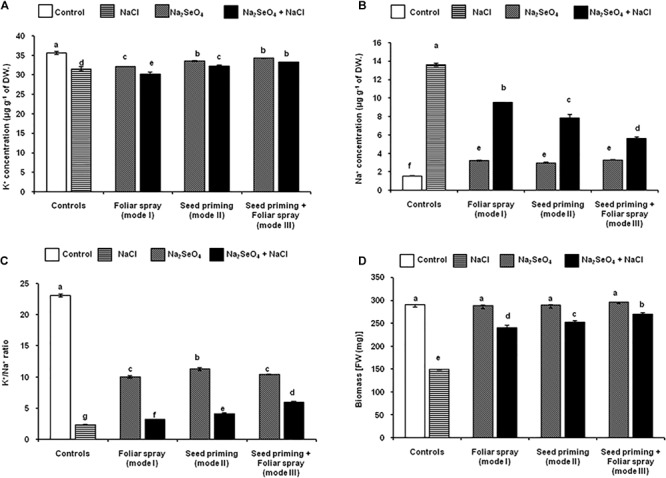
Effect of different modes of Na_2_SeO_4_ fortification on the changes in potassium (K^+^) and sodium (Na^+^) content, potassium/sodium (K^+^/Na^+^) ratio in the shoots and total fresh biomass accumulation. **(A)** K^+^ ion concentration (μg g^-1^ DW), **(B)** Na^+^ ion concentration (μg g^-1^ DW), **(C)** K^+^/Na^+^ ratio calculated from the data in Figure 3A,B, and **(D)** total fresh biomass accumulation [FW (mg)]. Bars represent the mean values of three independent experiments with standard errors. Means followed by different letters are significantly different according to Duncan’s multiple range test (DMRT) at 5% level.

A sharp increase in the level of Na^+^ and a significant decline in the level of K^+^ ions were observed when the plants were exposed to NaCl stress (Figure 3A,B). However, the plants supplemented with Na_2_SeO_4_ prior to exposure to NaCl accumulated a significantly lower level of Na^+^ ions and yielded a higher K^+^/Na^+^ ratio, relative to their control plants (treated with NaCl alone) (Figure 3A,C and Supplementary Table S1). A significantly higher K^+^/Na^+^ ratio (156.5%) was recorded for the plants that received Na_2_SeO_4_ by mode III (combination of seed priming and foliar spray) followed by mode II (77.7%) and mode I (36.7%) compared to control plants (without Na_2_SeO_4_ treatment) under NaCl stress (Figure 3C and Supplementary Table S1).

### Influence of Mode of Na_2_SeO_4_ Application on Biomass Accumulation

Clear phenotypic differences were observed between the control and salinity stressed rice plants. In the absence of Na_2_SeO_4,_ the salinity stressed control plants exhibited chlorosis, necrosis, and leaf burning. When plants were grown under optimal growth conditions (in the absence of NaCl), 6 mg l^-1^ Na_2_SeO_4_ treatment did not affect plant performance in terms of length, FW of roots and shoots, and total biomass. No visible phenotypic differences were observed between the control and 6 mg l^-1^ Na_2_SeO_4_ treated plants. Though no phenotypic variations were observed in the Na_2_SeO_4_ treated plants under the salinity stress, a significant reduction in the length and FW of roots and shoots, as well as total biomass were observed. However, the Na_2_SeO_4_ treated plants maintained a significantly higher length and FW of roots and shoots and total biomass compared to the control plants under salinity stress.

A substantial reduction (48.7%) in total biomass (FW) was observed for rice plants under NaCl stress compared to control plants (Figure 3D). However, 6 mg l^-1^ Na_2_SeO_4_ treatment significantly improved the total biomass of rice plants under NaCl stress (Figure 3D). When 6 mg l^-1^ Na_2_SeO_4_ was supplied, the total biomass was similar to that of the control plants (Figure 3D) indicating that 6 mg l^-1^ Na_2_SeO_4_ is not a lethal concentration for 2-week-old rice plants. However, the mode of exogenous Na_2_SeO_4_ supply strongly influenced the plant performance under NaCl stress. A significantly higher biomass was observed for the plants that received the Na_2_SeO_4_ by combined treatment (mode III, 82.1%) followed by plants developed from Na_2_SeO_4_ treated seeds (mode II, 73.9%), and plants sprayed with Na_2_SeO_4_ (mode I, 66.1%) compared to the control plants (without Na_2_SeO_4_) under NaCl stress (Figure 3D).

### Influence of Na_2_SeO_4_ on the Activities of Antioxidant Enzymes

**FIGURE 4:**
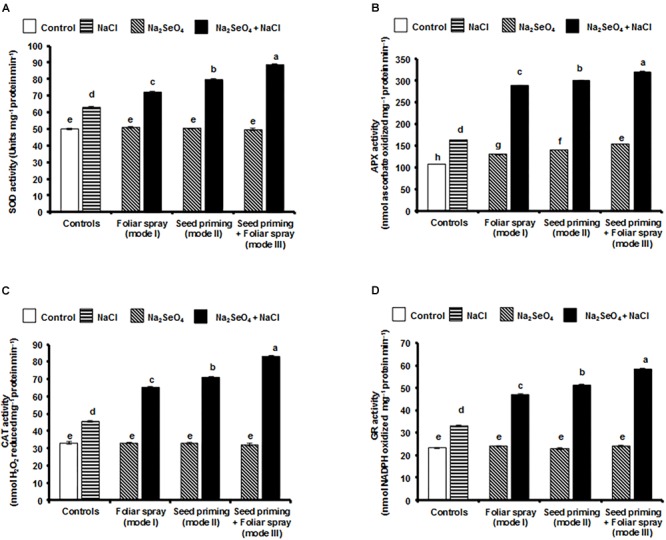
Effect of different modes of Na_2_SeO_4_ fortification on the activities of antioxidant enzymes. **(A)** superoxide dismutase (Units mg^-1^ protein min^-1^), **(B)** ascorbate peroxidase (nmol ascorbate oxidized mg^-1^ protein min^-1^), **(C)** catalase (nmol H_2_O_2_ reduced mg^-1^ protein min^-1^), and **(D)** glutathione reductase (nmol NADPH oxidized mg^-1^ protein min^-1^). Bars represent the mean values of three independent experiments with standard errors. Means followed by different letters are significantly different according to Duncan’s multiple range test (DMRT) at 5% level.

As shown in Figure 4A, no significant differences in SOD activity were observed between the control and Na_2_SeO_4_ treated plants under normal growth conditions. The SOD activity increased, when plants were exposed to NaCl stress. Plants treated with 6 mg l^-1^ Na_2_SeO_4_ maintained significantly higher SOD activity (Figure 4A) when grown in salinity stress conditions compared to control plants (without Na_2_SeO_4_) indicating that Na_2_SeO_4_ has the potential to enhance the SOD activity under salinity stress. Significantly higher SOD activity was observed in the plants that received the Na_2_SeO_4_ by mode III (40.7%) followed by mode II (24.9%) and mode I (14.2%) compared to the control plants (without Na_2_SeO_4_) under NaCl stress (Figure 4A).

The APX activity was higher in rice plants treated with either NaCl or 6 mg l^-1^ Na_2_SeO_4_ compared to control plants. Furthermore, the increase in enzyme activity was higher in plants enriched with Se prior to salinity stress (Figure 4B). The stimulatory effect of Na_2_SeO_4_ on the activity of APX was more evident in the presence of NaCl stress. Significantly higher APX activity was recorded in the plants receiving the Na_2_SeO_4_ by mode III (92.7%) followed by mode II (83.2%) and mode I (74.6%) compared to the control plants (without Na_2_SeO_4_) under NaCl stress (Figure 4B).

No significant differences in CAT activity were recorded for control and 6 mg l^-1^ Na_2_SeO_4_ treated plants in the absence of NaCl stress. When plants were exposed to NaCl stress, CAT activity increased significantly. However, Na_2_SeO_4_ treated plants showed a higher CAT activity than plants under NaCl stress (Figure 4C). Plants that received Na_2_SeO_4_ by mode III (combination of seed priming and foliar spray) showed significantly higher CAT activity (82.9%) followed by mode II (56.3%) and mode I (43.3%) compared to the control plants (without Na_2_SeO_4_) under NaCl stress (Figure 4C).

No substantial differences in the GR activity were observed between the control and Na_2_SeO_4_ treated plants in the absence of NaCl stress (Figure 4D). The NaCl treatment significantly improved the GR activity. However, when Na_2_SeO_4_ treated plants were exposed to NaCl stress, a significantly higher GR activity was observed compared to plants exposed to NaCl stress alone. The plants that received Na_2_SeO_4_ by mode III (combination of seed priming and foliar spray) showed a significantly higher GR activity (77.2%) followed by plants developed from Na_2_SeO_4_ treated seeds (mode II, 55.4%), and plants sprayed with Na_2_SeO_4_ (mode I, 42.7%) compared to the control plants (without Na_2_SeO_4_) under NaCl stress (Figure 4D).

**FIGURE 5:**
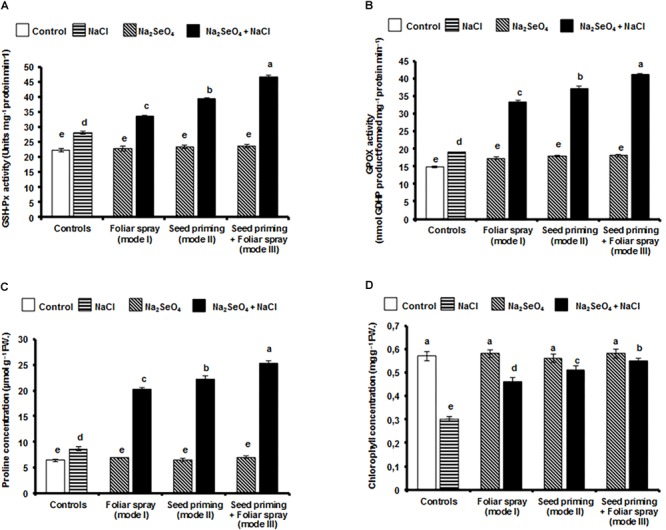
Effect of different modes of Na_2_SeO_4_ fortification on the activities of antioxidant enzymes and the proline and chlorophyll contents. **(A)** glutathione peroxidase (Units mg^-1^ protein min^-1^), **(B)** guaiacol peroxidase (nmol GDHP product formed mg^-1^ protein min^-1^), **(C)** proline (μmol g^-1^ FW), and **(D)** chlorophyll (mg g^-1^ FW). Bars represent the mean values of three independent experiments with standard errors. Means followed by different letters are significantly different according to Duncan’s multiple range test (DMRT) at 5% level.

Plants treated with NaCl showed increased GSH-Px activity. However, in the absence of NaCl, no significant increase was observed in the control plants or in the plants that received only Na_2_SeO_4_ (Figure 5A). The Na_2_SeO_4_ treated plants under NaCl stress showed significantly higher GSH-Px activity, compared with the plants subjected to NaCl stress without Na_2_SeO_4_ treatment. Significantly higher GSH-Px activity was observed in the plants that received the Na_2_SeO_4_ by mode III (66.1%) followed by mode II (40.1%) and mode I (19.3%) compared to the control plants (without Na_2_SeO_4_) under NaCl stress (Figure 5A).

The GPOX activity was elevated in rice plants treated with either NaCl or 6 mg l^-1^ Na_2_SeO_4_ compared with the control plants (Figure 5B). When Na_2_SeO_4_ treated plants were exposed to NaCl stress, significantly higher GPOX activity was observed than for plants exposed to NaCl stress alone. Plants that received Na_2_SeO_4_ by mode III (combination of seed priming and foliar spray) showed significantly higher GPOX activity (115.7%) followed by mode II (94.8%) and mode I (74.9%) compared to the control plants (without Na_2_SeO_4_) under NaCl stress (Figure 5B).

### Influence of Na_2_SeO_4_ on Proline Concentration

The concentration of free proline increased significantly in plants grown under NaCl stress (Figure 5C). However, no such increase was observed in the non-treated control or in the plants treated with Na_2_SeO_4_ alone. In Na_2_SeO_4_ treated plants exposed to NaCl stress, the proline concentration was significantly increased and was higher than for plants exposed to NaCl stress alone. The mode of Na_2_SeO_4_ supplementation influenced the proline concentration and plants that received Na_2_SeO_4_ by mode III (combination of seed priming and foliar spray) showed a higher proline content (191.1%) followed by mode II (156.4%) and mode I (133.3%) compared to control plants (without Na_2_SeO_4_) under NaCl stress (Figure 5C).

### Influence of Na_2_SeO_4_ on Chlorophyll Concentration

The application of Na_2_SeO_4_ did not influence the chlorophyll content in rice plants when plants were grown in the absence of NaCl stress. A reduction in chlorophyll content was observed in Na_2_SeO_4_ treated plants grown under NaCl stress. However, chlorophyll degradation was much lower than for plants not treated with Na_2_SeO_4_. Plants that received Na_2_SeO_4_ showed a significantly higher chlorophyll content compared to the plants under NaCl stress (Figure 5D). Among the different modes of Na_2_SeO_4_ supplementation, a higher chlorophyll concentration was observed in the plants that received Na_2_SeO_4_ by the combination of seed priming and foliar spray (83.3%) followed by individual treatments of seed priming (70%) and foliar spray (53.3%) compared to control plants (without Na_2_SeO_4_) under NaCl stress (Figure 5D).

### Influence of Na_2_SeO_4_ on Relative Water Content

**FIGURE 6:**
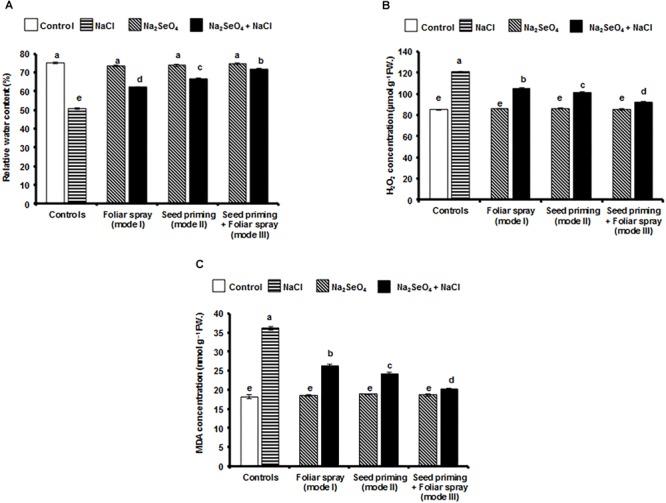
Effect of different modes of Na_2_SeO_4_ fortification on the performance of plants under NaCl stress. **(A)** relative water content (%), **(B)** hydrogen peroxide concentration (μmol g^-1^ FW), and **(C)** malondialdehyde concentration (nmol g^-1^ FW). Bars represent the mean values of three independent experiments with standard errors. Means followed by different letters are significantly different according to Duncan’s multiple range test (DMRT) at 5% level.

Under optimal growth conditions (in the absence of NaCl), the control as well as Na_2_SeO_4_ treated plants maintained a fairly high level of relative water content (Figure 6A). Plants showed a drastic reduction in RWC under NaCl stress. The Na_2_SeO_4_ treated plants maintained a significantly higher level of RWC. A significantly higher RWC was observed in the plants that received the Na_2_SeO_4_ by mode III (41.2%) followed by mode II (30.7%) and mode I (22.2%) compared to the control plants (without Na_2_SeO_4_) under NaCl stress (Figure 6A).

### Influence of Na_2_SeO_4_ on H_2_O_2_ Concentration

Control and Na_2_SeO_4_ treated plants accumulated less H_2_O_2_ when grown under the optimal growth conditions. An elevated level of H_2_O_2_ was observed in Na_2_SeO_4_ untreated plants, while a significantly lower concentration was observed in Na_2_SeO_4_ treated plants under NaCl stress (Figure 6B). Among the different modes of Na_2_SeO_4_ supplementation, a significantly lower level of H_2_O_2_ was observed in the plants that received Na_2_SeO_4_ by the combination of seed priming and foliar spray (23.5%) followed by individual treatments of seed priming (16%) and foliar spray (12.8%) compared to control plants (without Na_2_SeO_4_) under NaCl stress (Figure 6B).

### Influence of Na_2_SeO_4_ on MDA Concentration

The MDA concentration in control and Na_2_SeO_4_ treated plants grown under optimal growth conditions was similar (Figure 6C). However, NaCl stress increased the MDA concentration significantly. The plants treated with Na_2_SeO_4_ maintained a fairly low concentration of MDA compared to untreated plants. A significantly lower MDA level was observed in the plants that received the Na_2_SeO_4_ by mode III (79.5%) followed by mode II (49.7%) and mode I (37.7%) compared to control plants (without Na_2_SeO_4_) under NaCl stress (Figure 6C).

### Expression Analysis of *OsNHX1*

**FIGURE 7:**
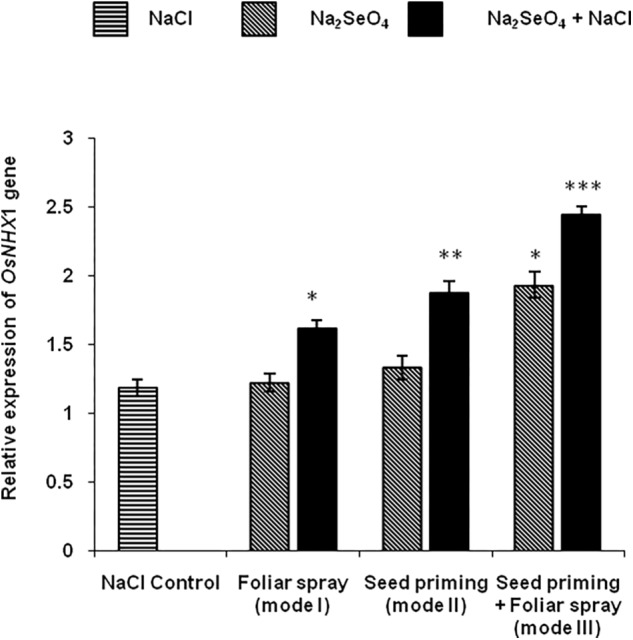
Quantitative real time-PCR analysis of OsNHX1 transcripts in the shoots of rice plants treated with Na_2_SeO_4_ and NaCl. Bars represent the mean values of three independent experiments with standard errors. Asterisks indicate statistically significant differences compared to control samples. ^∗^ indicates P value is <0.05; ^∗∗^ indicates P value is <0.01; ^∗∗∗^ indicates P value is <0.001.

*OsNHX*1 expression is significantly upregulated in plants that received Na_2_SeO_4_ prior to salinity stress compared to plants exposed to either NaCl or Na_2_SeO_4_ alone. Under salinity stress, the plants that received the Na_2_SeO_4_ both by the combination of seed priming and spraying (mode III) showed significantly higher transcript levels than the plants that received Na_2_SeO_4_ either by foliar spray (mode I) or seed priming (mode II) (Figure 7).

## Discussion

In modern agricultural practices, salinity is one of the most important abiotic stress factors limiting crop development and productivity. Under salinity conditions molecular oxygen (O_2_) acts as an electron acceptor, resulting in the accumulation of ROS such as singlet oxygen (^1^O_2_), the hydroxyl radical (OH^-^), the superoxide radical (O^-2^), and H_2_O_2_. These ROS are strongly oxidizing compounds and hence disturb plant cell integrity. It has been reported that antioxidant compounds can detoxify ROS induced by salinity stress. Salinity tolerance is positively interrelated with the activity of antioxidant enzymes, such as SOD, APX, CAT, GSH-Px, GR and with the accumulation of non-enzymatic antioxidant compounds. It has been proven that, at a lower concentration, Na_2_SeO_4_ acts as an antioxidant and protects the plants from ROS induced oxidative damage. However, at higher concentrations, Na_2_SeO_4_ acts as prooxidant and induces the formation of ROS and causes oxidative stress (Hartikainen et al., 2000). Hence, selection of the optimum concentration of Na_2_SeO_4_ is a crucial step before applying this compound on rice plants. In the present study, 6 mg l^-1^ Na_2_SeO_4_ was proven the optimal concentration for treatment of 2-week-old rice plants. Beyond 6 mg l^-1^ plants showed necrotic symptoms under 150 mM NaCl stress.

Different methods have been reported to fortify plants with antioxidant compounds against abiotic stresses and among them seed priming and foliar spray are considered as the most promising methods. Seed priming has emerged as an effective and sensible approach for increasing stress tolerance in two steps. First, seed priming triggers many germination associated activities such as enhanced energy metabolism, embryo expansion, and endosperm weakening (Ibrahim, 2016). Second, priming imposes an abiotic stress on seeds that represses radicle protrusion, but stimulates stress responses, inducing cross-tolerance, enzyme activation, build-up of germination enhancing metabolites, metabolic repair during imbibition, and osmotic adjustment (Hussain et al., 2016). These two strategies together make up a “priming memory” in the seeds, which can be employed upon later stress exposure and mediates the greater stress tolerance of germinating primed seeds (Bruce et al., 2007; Chen and Arora, 2013; Pastor et al., 2013). Foliar application is considered as an effective method to provide essential nutrients, organic acids, and several growth regulators to plants for their normal growth and development (Wojcik, 2004). During foliar application, plants are able to absorb essential nutrients through their stomata and leaf epidermis.

In our study seed priming and foliar spray considerably increased the Se concentration in the rice plants either in presence or absence of salinity stress (Figure 2B). The Se content in the untreated control rice plants was lower than in the treated plants. The presence of Se in the untreated control rice plants is attributed to the Se native to the soil used to grow the plants and traces of Se in the solution used for fertilization. The concentration of Se in the plants was also influenced by the mode of Se application, and the presence or absence of salinity stress. Plants that received Na_2_SeO_4_ by seed priming and foliar spray accumulated Se more effectively compared to plants receiving the individual treatments. The salinity stressed plants accumulated higher Se than the unstressed plants. The higher Se concentration in the stressed rice plants indicates that the stress effects were severe and Se is required to enhance the ROS scavenging activity, reduce MDA concentration and membrane damage. Seed priming and foliar spray may also increase the concentration of proline and antioxidant enzyme activities which are necessary to increase crop resistance against salinity stress (Kazemi and Eskandari, 2012; Kubala et al., 2015a,b; Shalaby et al., 2017). Irrespective of the mode of Se application, the total biomass, the antioxidant enzyme activities and the concentration of proline increased to a notable level in Na_2_SeO_4_ treated plants under salinity stress (Figure 3D, 4A–D, 5A–C). However, the combination of seed priming and foliar spray is more effective than the single treatments and ultimately resulted in higher biomass, antioxidant enzyme activities, and proline content (Figure 3D, 4A–D, 5A–C). These data suggest that the Na_2_SeO_4_ supplied by foliar spray to the plants developed from the Se primed seeds acted as a booster dose to enhance the antioxidant enzyme activities and proline levels after NaCl stress.

It is well known that plants under saline conditions, accumulate more Na^+^ ions, which disturbs the ionic balance, plant metabolism, and induce oxidative damage. Plant tolerance toward salinity depends on the K^+^ ion status in the plant tissues (Hasegawa et al., 2000). In the present study, salinity marginally affected the K^+^ ion content, but increased the Na^+^ content in leaves, substantially lowering the K^+^/Na^+^ ratio. The plants that were treated with Na_2_SeO_4_ showed a lower Na^+^ concentration and a higher K^+^/Na^+^ ratio than the untreated control plants under the salinity stress conditions. Na_2_SeO_4_ treatment by the combination of seed priming and foliar spray (mode III) is more effective than the individual treatments in maintaining higher K^+^/Na^+^ ratios. The obtained results were consistent with the findings of Astaneh et al. (2017) and Shekari et al. (2017) reported for garlic and dill, respectively, who observed that Se reduced the accumulation of Na^+^ ions, leading to an increase of the K^+^/Na^+^ ratio compared to the respective untreated control plants.

To investigate the mechanism by which Se reduces the accumulation of sodium ions and their toxic effects on plant growth and development, we analyzed the transcript levels for *OsNHX*1, known as an important vacuolar Na^+^/H^+^ antiporter that catalyzes the sequestration of Na^+^ ions into the root and shoot vacuoles (Almeida et al., 2017). The sequestration of Na^+^ ions into the root vacuoles minimizes the interference in the water movement to the above ground parts of the plants and maintains the osmotic balance. Tomato, *Brassica napus*, and poplar plants overexpressing the *NHX*1 gene showed significant resistance to salinity stress due to the sequestration of Na^+^ ions into the vacuoles (Zhang and Blumwald, 2001; Zhang et al., 2001; Jiang et al., 2012). *OsNHX*1 transcript levels were significantly higher in Na_2_SeO_4_ treated plants under NaCl stress. The plants that received the Se by the combination of seed priming and foliar spray accumulated higher amounts of Se ions and exhibited significantly higher *OsNHX*1 transcript levels (Figure 7). It can be envisaged that higher transcript levels for *OsNHX*1 contribute to the sequestration of higher concentrations of Na^+^ ions into the root vacuoles and reduced the transportation of Na^+^ ions to the shoots. This could be the reason why the shoots from the plants that received Na_2_SeO_4_ by the combination of seed priming and foliar spray accumulated low levels of Na^+^ ions compared to the plants receiving the individual treatments under NaCl stress.

Salinity stress is accompanied with a robust accumulation of ROS, and hampers plant growth and development. Recently, it was shown that exogenous supplementation of Na_2_SeO_4_ acts as an antioxidant, enhancing the activity of antioxidant enzymes such as SOD, APX and CAT, and protects the plants from various environmental stress conditions (Djanaguiraman et al., 2010; Hasanuzzaman and Fujita, 2011; Hasanuzzaman et al., 2011; Wang, 2011; Malik et al., 2012; Proietti et al., 2013; Iqbal et al., 2015). When plants treated with Na_2_SeO_4_ by both seed priming and foliar spray (mode III) were exposed to salinity stress, the SOD, APX, and CAT activities were higher than in the control plants (40.7, 92.7, and 82.9%, respectively) and protected the plants from ROS induced oxidative damage. The results were in agreement with previous reports where, exogenous Na_2_SeO_4_ treatment significantly improved the SOD, APX, and CAT activity in salinity stressed seedlings of rapeseed and *Anethum graveolens* (Hasanuzzaman et al., 2011; Shekari et al., 2017). Recently it has been reported that Se treatment significantly enhanced the translocation of minerals such as iron, zinc, and manganese into the shoots of rice (Moulick et al., 2018). These minerals are integral components of antioxidant enzymes and enhance the activities of SOD, POD, and CAT (Nouet et al., 2011).

Jiang et al. (2017) reported that exogenous supply of Se to maize plants under salinity stress resulted in the upregulation of genes involved in the antioxidant defense. Priming the maize plants with Se significantly upregulated the expression of mitogen activated protein kinase (*MAPK5* and *MAPK7*), and calcium dependent protein kinase (*CPK11*) genes, and the up-regulation of these genes contributed to Se-induced antioxidant defense system (Jiang et al., 2017). The *MAPK* cascade is at the center of cell signal transduction and has been reported to be involved in stress-related signaling pathways (Liu et al., 2018). Salinity stress induces the accumulation of ABA (Sripinyowanich et al., 2013). ABA-induced H_2_O_2_ production activates *MAPK*, which in turn induces the expression and activities of antioxidant enzymes (Zhang et al., 2006).

Glutathione peroxidase and glutathione reductase are important enzymes and play a vital role in scavenging H_2_O_2_ and lipid peroxides to water and lipid alcohols, respectively (Djanaguiraman et al., 2010; Hasanuzzaman and Fujita, 2011; Feng et al., 2013). GSH-Px is believed to be a key enzyme, which can be widely and robustly activated by Se in various plants exposed to several environmental stresses (Feng et al., 2013). In the presence of selenium, H_2_O_2_ is primarily and majorly quenched by GSH-Px and then APX, CAT, and GR eliminate the remnants of H_2_O_2_. Irrespective of the mode of Na_2_SeO_4_ application, the Se treatment significantly increased the GSH-Px and GR activities compared to untreated plants under salinity stress conditions. When plants treated with Na_2_SeO_4_ by both seed priming and foliar spray (mode III) were exposed to salinity stress, the GSH-Px and GR activities were higher than in the control plants (66.1 and 77.2%, respectively). The higher activity of GSH-Px in these plants might be due to the higher level of Se which is necessary for the formation of selenocysteine. Selenocysteine is present at the catalytic site of GSH-Px, and Se availability regulates GSH-Px activity (Feng et al., 2013). The increased GSH-Px and GR activity brought down the levels of H_2_O_2_ and MDA (Figure 6B,C) and protected the salinity stressed rice plants from ROS induced oxidative damages. These results are in agreement with Hasanuzzaman et al. (2011), who observed elevated levels of GSH-Px and GR in Na_2_SeO_4_ treated rapeseed seedlings under salinity stress conditions.

In response to salinity stress, plants accumulate large quantities of different types of compatible solutes. These solutes are low molecular weight, non-toxic soluble organic compounds that provide plants protection against salinity stress by contributing to ROS detoxification, protection of membrane integrity, and protein stabilization (Subramanyam et al., 2011). Proline is one of the important compatible solutes that is robustly accumulated in plants during several abiotic stresses and protects plants from ROS induced toxicity. Proline also acts as a molecular chaperone which is able to protect protein integrity (Szabados and Savouré, 2010). Proline contains chelating capacity and binds with metal ions which may serve as a defense mechanism in stressed plants (Nawaz et al., 2015). It has been reported that salinity stress interferes with the assimilation, accumulation, and metabolism of nitrogen which is an important element in the biosynthesis of proline (Ullrich, 2002). It is reported that a low concentration of Na_2_SeO_4_ is beneficial for plants in increasing the proline concentration under various abiotic stresses (Hawrylak-Nowak, 2009; Nawaz et al., 2015) by enhancing the nitrate reductase activity and nitrogen content (Khan et al., 2015). The results of the present study also indicated that application of Na_2_SeO_4_ increased proline in salinity stressed plants. The plants that received Na_2_SeO_4_ by both seed priming and foliar spray (mode III) prior to salinity stress showed the highest proline concentration (191.1%) than the control plants. The high proline concentration in these plants might be caused by the higher Se content (Figure 2B). The higher amount of Se might be responsible to lower the sodium ion accumulation (Figure 3B) in the rice plants and thereby enhances nitrate reductase activity necessary for proline biosynthesis. The results agree with previous reports where, Na_2_SeO_4_ pre-treated canola plants (Hashem et al., 2013) and cucumber seedlings (Hawrylak-Nowak, 2009) showed a higher proline content under NaCl induced salinity stress.

Salinity stress causes photo-oxidative reactions, which in turn cause rapid and large accumulation of superoxide radicals and H_2_O_2_ content. The higher level of superoxide radicals and H_2_O_2_ degrades the membranes of thylakoids and chloroplasts which ultimately leads to chlorophyll degradation (Subramanyam et al., 2011). It is known that chlorophyll degradation is directly proportional to salinity stress resistance and salinity stress resistant plants maintain significantly higher chlorophyll levels. Moulick et al. (2018) reported that Se treatment significantly enhanced the accumulation of manganese, zinc, and iron. Manganese is involved in the elimination of ROS by enhancing the antioxidant enzymes, while zinc restore ROS-induced damage in photosynthetic apparatus and prevent the chlorophyll degradation (Chen et al., 2008; Carvalho et al., 2014). Iron is an important element in the chlorophyll biosynthesis and photosynthesis (Nouet et al., 2011). In the present study, a lower level of chlorophyll degradation was observed in the plants that received Na_2_SeO_4_ by both seed priming and foliar spray (mode III) compared to individual treatments under the salinity stress. The results indicate that the exogenous Na_2_SeO_4_ efficiently scavenged the ROS by increasing the antioxidant enzyme activities (Figure 4A–D, 5A,B), thereby reducing the damage of chloroplasts and increasing the chlorophyll content. Previous reports showed that Na_2_SeO_4_ treatment reduced the chlorophyll degradation in salinity stressed cucumber, canola, parsley, and garlic (Hawrylak-Nowak, 2009; Hashem et al., 2013; Habibi, 2017; Astaneh et al., 2017).

It is a well-known fact that salinity stress reduces root hydraulic conductivity and functionality of aquaporins, and minimizes the transport of water to the above ground parts (López-Berenguer et al., 2006). The RWC is considered as an important parameter for salinity tolerance (Malatrasi et al., 2002; Rampino et al., 2006; Talame et al., 2007). Maintenance of a high water level is an indication for high salinity survival (Flower and Ludlow, 1986). In the present study, the plants that received Na_2_SeO_4_ by the combination of seed priming and foliar spray (mode III) showed a higher RWC under salinity stress. The higher RWC in these plants might be due to the higher expression levels for *OsNHX*1 (Figure 7) which is involved in the sequestration of Na^+^ ions into the root vacuoles. The higher levels of Na^+^ ions in the salinity stressed plants cause sodicity, which reduces root growth and reduces the water movement through the root with a decrease in hydrolic conductivity, resulting in a lower RWC in the plants (Rengasamy and Olsson, 1993). In the current study, Na_2_SeO_4_ treated plants accumulated lower levels of Na^+^ ions (Figure 3B) and exhibited better root growth (Figure 1A,C) than the control plants (grown in the absence of Na_2_SeO_4_), which might have favored water movement to the shoots and maintained higher RWC under salinity stress (Figure 6A). Similarly, Na_2_SeO_4_ pre-treatment in garlic plantlets, rapeseed, and cucumber seedlings significantly improved the RWC under the salinity stress (Hawrylak-Nowak, 2009; Hasanuzzaman et al., 2011; Astaneh et al., 2017). Recently, Fu et al. (2018) reported drastic differences in the absolute expression levels of SOS, HKT, and NHX family genes between genotypes of barley and rice, and suggested that the differential expression of Na and K transporters accounts for the differences in Na/K homeostasis and salt tolerance between cereals. More in depth experiments are needed to fully elucidate the mode of action of sodium selenate.

Salinity induced oxidative stress triggers the accumulation of higher levels of H_2_O_2_ causing apoptosis, cell shrinkage, chromatin condensation, and DNA fragmentation (Houot et al., 2001). A variety of enzymatic and non-enzymatic antioxidants play a crucial role in eliminating H_2_O_2_ (Gill and Tuteja, 2010). A significantly lower concentration of H_2_O_2_ was observed in the rice plants that received Na_2_SeO_4_ by both seed priming and foliar spray (mode III). The accumulation of lower amounts of H_2_O_2_ (Figure 6B) in these plants might be due to the elevated levels of APX and CAT (Figure 4B,C). The results were in agreement with previous reports where, Na_2_SeO_4_ pre-treated rapeseed seedlings (Hasanuzzaman et al., 2011) and canola plantlets (Hashem et al., 2013) showed lower levels of H_2_O_2_ under salinity stress.

Elevated levels of ROS production during salinity stress lead to the peroxidation of lipids present in the thylakoid membrane. Lipid peroxidation of plant cells was measured by the MDA concentration which is considered as a potent indicator of lipid peroxidation (Farmer and Mueller, 2013). The MDA levels in salt stressed rice were consistently higher, compared to the control plants. However, irrespective of the mode of application, Na_2_SeO_4_ treatment of rice plantlets prior to the NaCl stress significantly lowered the MDA content. The plants that received Na_2_SeO_4_ by mode III showed a significantly lower MDA concentration compared to the untreated control plants under NaCl stress indicating that Na_2_SeO_4_ played an important role in lowering the lipid peroxidation by improving the antioxidant enzymes and protecting the membranes (Pennanen et al., 2002; Feng et al., 2013). The results are also supported by several other reports where, Na_2_SeO_4_ treatment significantly reduced the MDA concentration in salinity stressed cucumber, rapeseed, and dill (Hawrylak-Nowak, 2009; Hasanuzzaman et al., 2011; Shekari et al., 2017).

After submission of this manuscript multiple publications described the beneficial effects of Se on abiotic stress tolerance in rice. Se application protects rice plants from water deficit stress (Andrade et al., 2018), since rice plants treated with Se showed a higher net photosynthesis, water use efficiency and antioxidant activities. Moulick et al. (2018) reported the influence of Se in mitigating arsenic induced phytotoxicity in rice. They reported that Se enhanced the translocation of minerals such as iron, zinc, and manganese into shoots and restricted the translocation of arsenic into the shoots developed from the Se primed seeds. Furthermore, Se treatment enhanced the germination rate, length of root and shoot, chlorophyll content, and reduced the accumulation of H_2_O_2_. Similarly, foliar spraying with Se, and mixture of silicon and Se also decreased the accumulation of cadmium in rice (Gao et al., 2018).

In conclusion, NaCl stress increased oxidative stress and severely affected rice growth and total biomass. The results of our study clearly showed the ability of Na_2_SeO_4_ to improve rice tolerance to NaCl stress. The growth mitigating effect of Na_2_SeO_4_ on rice plants grown at high salinity could be endorsed to the accumulation of proline and higher antioxidant enzyme activities. The combination of seed priming and foliar spray emerged as a best method to fortify the rice plants with Na_2_SeO_4_ to counteract the negative effects of salinity stress. Our experiments were not primarily designed to obtain new mechanistic insights. However, the new information that Na_2_SeO_4_ supplementation by the combination of seed priming and foliar spray is better than the individual treatment to alleviate the salinity stress in rice, will certainly help to implement similar strategies for other cereal crops against several abiotic stresses.

## Author Contributions

KS and EV conceived and designed the experiments. KS performed the experiments and analyzed the data. GD and KS performed the analyses. KS and EV contributed to the writing of the manuscript, and performed the final editing of the manuscript.

## Conflict of Interest Statement

The authors declare that the research was conducted in the absence of any commercial or financial relationships that could be construed as a potential conflict of interest.

## Supplementary Material

FILE S1 Selection of optimal concentration of sodium selenate (Na_2_SeO_4_).

FILE S2 Schematic representation of the experimental setup for control samples.

FILE S3 Schematic representation of Na_2_SeO_4_ application by mode I (foliar spray) to alleviate NaCl stress.

FILE S4 Schematic representation of Na_2_SeO_4_ application by mode II (seed priming) and mode III (combination of seed priming and foliar spray) to alleviate NaCl stress.

FILE S5 List of primers used for quantitative real time-PCR.

TABLE S1 Analysis of selenium, sodium, and potassium concentrations in sodium selenate (Na_2_SeO_4_) treated rice plant material using inductively coupled plasma–MS (ICP-MS) and inductively coupled plasma optical emission spectrophotometer (ICP-OES).

## References

[B1] Almeida D. M. Gregorio G. B. Oliveira M. M. Saibo N. J. M. (2017). Five novel transcription factors as potential regulators of OsNHX1 gene expression in a salt tolerant rice genotype. *Plant Mol. Biol.* 93 61–77. 10.1007/s11103-016-0547-7 27766460

[B2] Andrade F. R. da Silva G. N. Guimarães K. C. Barreto H. B. F. de Souza K. R. D. Guilherme L. R. G. (2018). Selenium protects rice plants from water deficit stress. *Ecotoxicol. Environ. Saf.* 164 562–570. 10.1016/j.ecoenv.2018.08.022 30149355

[B3] Arnon D. I. (1949). Copper enzymes in isolated chloroplasts. Polyphenol oxidase in *Beta vulgaris*. *Plant Physiol.* 24 1–15. 10.1104/pp.24.1.1 16654194 PMC437905

[B4] Arun M. Radhakrishnan R. Ai T. N. Naing A. H. Lee I. J. Kim C. K. (2016). Nitrogenous compounds enhance the growth of *Petunia* and reprogram biochemical changes against the adverse effect of salinity. *J. Hortic. Sci. Biotechnol.* 91 562–572. 10.1080/14620316.2016.1192961

[B5] Astaneh R. K. Bolandnazar S. Nahandi F. Z. Oustan S. (2017). The effects of selenium on some physiological traits and K, Na concentration of garlic (Allium sativum L.) under NaCl stress. *Inform. Proc. Agri.* 5 156–161. 10.1016/j.inpa.2017.09.003

[B6] Bates L. S. Waldren R. P. Teare I. D. (1973). Rapid determination of free proline for water-stress studies. *Plant Soil* 39 205–207. 10.1007/BF00018060 20688380

[B7] Bradford M. M. (1976). Rapid and sensitive method for the quantification of microgram quantities of protein utilizing the principle of protein-dye binding. *Anal. Biochem.* 72 248–254. 10.1016/0003-2697(76)90527-3 942051

[B8] Broadley M. R. Alcock J. Alford J. Cartwright P. Foot I. Fairweather-Tait S. J. (2010). Selenium biofortification of high-yielding winter wheat (*Triticum aestivum* L.) by liquid or granular Se fertilisation. *Plant Soil* 332 5–18. 10.1007/s11104-009-0234-4

[B9] Bruce T. Matthes M. C. Napier J. A. Pickett J. A. (2007). Stressful memories of plants: evidence and possible mechanisms. *Plant Sci.* 173 603–608. 10.1016/j.plantsci.2007.09.002

[B10] Carvalho E. R. Oliveira J. A. Pinho É. V. R. V. Neto J. C. (2014). Enzyme activity in soybean seeds produced under foliar application of manganese. *Ciênc. Agrotec. Lavras* 38 317–327. 10.1590/S1413-70542014000400001

[B11] Chen K. Arora R. (2013). Priming memory invokes seed stress-tolerance. *Environ. Exp. Bot.* 94 33–45. 10.1016/j.envexpbot.2012.03.005 23973468

[B12] Chen W. Yang X. He Z. Feng Y. Hu F. (2008). Differential changes in photosynthetic capacity, K chlorophyll fluorescence and chloroplast ultra structure between Zn efficient and Zn-inefficient rice genotypes (*Oryza sativa*) under low zinc stress. *Physiol. Plant* 132 89–101. 10.1111/j.1399-3054.2007.00992.x 18251873

[B13] Chinnusamy V. Jagendorf A. Zhu J. K. (2005). Understanding and improving salt tolerance in plants. *Crop. Sci.* 45 437–448. 10.2135/cropsci2005.0437

[B14] Djanaguiraman M. Prasad P. Seppanen M. (2010). Selenium protects sorghum leaves from oxidative damage under high temperature stress by enhancing antioxidant defense system. *Plant Physiol. Biochem.* 48 999–1007. 10.1016/j.plaphy.2010.09.009 20951054

[B15] Elia A. C. Galarini R. Taticchi M. I. Dorr A. J. M. Mantilacci L. (2003). Antioxidant responses and bioaccumulation in *Ictalurusmelas* under mercury exposure. *Ecotoxicol. Environ. Saf.* 55 162–167. 10.1016/S0147-6513(02)00123-9 12742363

[B16] Farmer E. E. Mueller M. J. (2013). ROS-mediated lipid peroxidation and RES-activated signaling. *Annu. Rev. Plant Biol.* 64 429–450. 10.1146/annurev-arplant-050312-120132 23451784

[B17] Feng R. Wei C. Tu S. (2013). The roles of selenium in protecting plants against abiotic stresses. *Environ. Exp. Bot.* 87 58–68. 10.1016/j.envexpbot.2012.09.002 29966265

[B18] Filek M. Keskinen R. Hartikainen H. Szarejko I. Janiak A. Miszalski Z. (2008). The protective role of selenium in rape seedlings subjected to cadmium stress. *J. Plant Physiol.* 165 833–844. 10.1016/j.jplph.2007.06.006 17913288

[B19] Flower D. J. Ludlow M. M. (1986). Contribution of osmotic adjustment to the dehydration tolerance of water-stressed pigeon pea [*Cajanus cajan* (L.) Mill sp.] leaves. *Plant Cell Environ.* 9 33–40. 10.1111/1365-3040.ep11589349

[B20] Foyer C. Halliwell B. (1976). The presence of glutathione and glutathione reductase in chloroplasts: a proposed role in ascorbic acid metabolism. *Planta* 133 21–25. 10.1007/BF00386001 24425174

[B21] Fu L. Shen Q. Kuang L. Yu J. Wu D. Zhang G. (2018). Metabolite profiling and gene expression of Na/K transporter analyses reveal mechanisms of the difference in salt tolerance between barley and rice. *Plant Physiol. Biochem.* 130 248–257. 10.1016/j.plaphy.2018.07.013 30021179

[B22] Gao M. Zhou J. Liu H. Zhang W. Hu Y. Liang J. (2018). Foliar spraying with silicon and selenium reduces cadmium uptake and mitigates cadmium toxicity in rice. *Sci. Total Environ.* 63 1100–1108. 10.1016/j.scitotenv.2018.03.047 29727936

[B23] Germ M. Kreft I. Osvald J. (2005). Influence of UV-B exclusion and selenium treatment on photochemical efficiency of photosystem II, yield and respiratory potential in pumpkins (*Cucurbita pepo* L.). *Plant Physiol. Phytochem.* 43 445–448. 10.1016/j.plaphy.2005.03.004 15949721

[B24] Gill S. S. Tuteja N. (2010). Reactive oxygen species and antioxidant machinery in abiotic stress tolerance in crop plants. *Plant Physiol. Biochem.* 48 909–930. 10.1016/j.plaphy.2010.08.016 20870416

[B25] Grattan S. R. Zeng L. Shannon M. C. Roberts S. R. (2002). Rice is more sensitive to salinity than previously thought. *Calif. Agric.* 56 189–195.

[B26] Habibi G. (2017). Selenium ameliorates salinity stress in *Petroselinum crispum* by modulation of photosynthesis and by reducing shoot Na accumulation. *Russ. J. Plant Physiol.* 64 368–374. 10.1134/S1021443717030086

[B27] Hartikainen H. Xue T. (1999). The promotive effect of selenium on plant growth as triggered by ultraviolet irradiation. *J. Environ. Q.* 28 1272–1275. 10.2134/jeq1999.00472425002800040043x

[B28] Hartikainen H. Xue T. Piironen V. (2000). Selenium as an anti-oxidant and pro-oxidant in ryegrass. *Plant Soil* 225 193–200. 10.1023/A:1026512921026

[B29] Hasanuzzaman M. Alam M. M. Rahman A. Hasanuzzaman M. Nahar K. Fujita M. (2014). Exogenous proline and glycine betaine mediated upregulation of antioxidant defense and glyoxalase systems provides better protection against salt-induced oxidative stress in two rice (*Oryza sativa* L.) varieties. *Biomed. Res. Int.* 2014:757219. 10.1155/2014/757219 24991566 PMC4065706

[B30] Hasanuzzaman M. Fujita M. (2011). Selenium pretreatment upregulates the antioxidant defense and methylglyoxal detoxification system and confers enhanced tolerance to drought stress in rapeseed seedlings. *Biol. Trace Elem. Res.* 143 1758–1776. 10.1007/s12011-011-8998-9 21347652

[B31] Hasanuzzaman M. Hossain M. A. Fujita M. (2011). Selenium-induced up-regulation of the antioxidant defense and methylglyoxal detoxification system reduces salinity-induced damage in rapeseed seedlings. *Biol. Trace Elem. Res.* 143 1704–1721. 10.1007/s12011-011-8958-4 21264525

[B32] Hasegawa P. M. Bressan R. A. Zhu J. K. Bohnert H. J. (2000). Plant cellular and molecular responses to high salinity. *Annu. Rev. Plant Physiol. Plant Mol. Biol.* 51 463–499. 10.1146/annurev.arplant.51.1.463 15012199

[B33] Hashem H. A. Hassanein R. A. Bekheta M. A. El –Kady F. A. (2013). Protective role of selenium in canola (*Brassica napus* L.) plant subjected to salt stress. *Egypt. J. Exp. Biol.* 9 199–211.

[B34] Hawrylak-Nowak B. (2009). Beneficial effects of exogenous selenium in cucumber seedlings subjected to salt stress. *Biol. Trace Elem. Res.* 132 259–269. 10.1007/s12011-009-8402-1 19434374

[B35] Heath R. L. Packer L. (1968). Photoperoxidation in isolated chloroplasts. Kinetics and stoichiometry of fatty acid peroxidation. *Arch. Biochem. Biophys.* 125 189–198. 10.1016/0003-9861(68)90654-1 5655425

[B36] Hellemans J. Mortier G. De Paepe A. Speleman F. Vandesompele J. (2007). qBase relative quantification framework and software for management and automated analysis of real-time quantitative PCR data. *Genome Biol.* 8:R19. 17291332 10.1186/gb-2007-8-2-r19 PMC1852402

[B37] Houot V. Etienne P. Petitot A. S. Barbier S. Blein J. P. Suty L. (2001). Hydrogen peroxide induces programmed cell death features in cultured tobacco BY-2 cells, in a dose-dependent manner. *J. Exp. Bot.* 52 1721–1730. 11479338

[B38] Hussain S. Khan F. Hussain H. A. Nie L. (2016). Physiological and biochemical mechanisms of seed priming-induced chilling tolerance in rice cultivars. *Front. Plant Sci.* 7:116. 10.3389/fpls.2016.00116 26904078 PMC4746480

[B39] Ibrahim E. A. (2016). Seed priming to alleviate salinity stress in germinating seeds. *J. Plant Physiol.* 192 38–46. 10.1016/j.jplph.2015.12.011 26812088

[B40] Iqbal M. Hussain I. Liaqat H. Ashraf M. A. Rasheed R. Rehman A. U. (2015). Exogenously applied selenium reduces oxidative stress and induces heat tolerance in spring wheat. *Plant Physiol. Biochem.* 94 95–103. 10.1016/j.plaphy.2015.05.012 26057700

[B41] Jiang C. Zheng Q. Liu Z. Xu W. Liu L. Zhao G. (2012). Overexpression of *Arabidopsis thaliana* Na+/H+ antiporter gene enhanced salt resistance in transgenic poplar (*Populus* × *euramericana* ‘Neva’). *Trees* 26 685–694. 10.1007/s00468-011-0635-x

[B42] Jiang C. Zu C. Lu D. Zheng Q. Shen J. Wang H. (2017). Effect of exogenous selenium supply on photosynthesis, Na+ accumulation and antioxidative capacity of maize (*Zea mays* L.). *Sci. Rep.* 7:42039. 10.1038/srep42039 28169318 PMC5294586

[B43] Kasote D. M. Katyare S. S. Hegde M. V. Bae H. (2015). Significance of antioxidant potential of plants and its relevance to therapeutic applications. *Int. J. Biol. Sci.* 11 982–991. 10.7150/ijbs.12096 26157352 PMC4495415

[B44] Kazemi K. Eskandari H. (2012). Does priming improve seed performance under salt and drought stress? *J. Basic Appl. Sci. Res.* 2 3503–3507.

[B45] Khan M. I. R. Nazirm F. Asgher M. Per T. S. Khan N. A. (2015). Selenium and sulfur influence ethylene formation and alleviate cadmium-induced oxidative stress by improving proline and glutathione production in wheat. *J. Plant Physiol.* 173 9–18. 10.1016/j.jplph.2014.09.011 25462073

[B46] Khatun S. Flowers T. J. (1995). Effects of salinity on seed set in rice. *Plant Cell Environ.* 18 61–67. 10.1111/j.1365-3040.1995.tb00544.x

[B47] Khush G. S. (2013). Strategies for increasing the yield potential of cereals: case of rice as an example. *Plant Breed.* 132 433–436. 10.1111/pbr.1991

[B48] Kubala S. Garnczarska M. Wojtyla L. Clippe A. Kosmala A. Zmienko A. (2015a). Deciphering priming-induced improvement of rapeseed (*Brassica napus* L.) germination through an integrated transcriptomic and proteomic approach. *Plant Sci.* 231 94–113. 10.1016/j.plantsci.2014.11.008 25575995

[B49] Kubala S. Wojtyla L. Quinet M. Lechowska K. Lutts S. Garnczarska M. (2015b). Enhanced expression of the proline synthesis gene P5CSA in relation to seed osmo priming improvement of *Brassica napus* germination under salinity stress. *Plant Sci.* 183 1–12. 10.1016/j.jplph.2015.04.009 26070063

[B50] Liu J. Wang J. Lee S. Wen R. (2018). Copper-caused oxidative stress triggers the activation of antioxidant enzymes via ZmMPK3 in maize leaves. *PLoS One* 13:e0203612. 10.1371/journal.pone.0203612 30222757 PMC6141078

[B51] Lobanov A. V. Hatfield D. L. Gladyshev V. N. (2008). Reduced reliance on the trace element selenium during evolution of mammals. *Genome Biol.* 9:R62. 10.1186/gb-2008-9-3-r62 18377657 PMC2397514

[B52] López-Berenguer C. García-Viguera C. Carvajal M. (2006). Are root hydraulic conductivity responses to salinity controlled by aquaporins in broccoli plants? *Plant Soil* 279 13–23. 10.1007/s11104-005-7010-x

[B53] Malatrasi M. Close T. J. Marmiroli N. (2002). Identification and mapping of a putative stress response regulator gene in barley. *Plant Mol. Biol.* 50 143–152. 10.1023/A:1016051332488 12139005

[B54] Malik J. A. Goel S. Kaur N. Sharma S. Singh I. Nayyar H. (2012). Selenium antagonises the toxic effects of arsenic on mungbean (*Phaseolus aureus* Roxb.) plants by restricting its uptake and enhancing the antioxidative and detoxification mechanisms. *Environ. Exp. Bot.* 77 242–248. 10.1016/j.envexpbot.2011.12.001

[B55] Moulick D. Santra S. C. Ghosh D. (2018). Seed priming with Se mitigates As-induced phytotoxicity in rice seedlings by enhancing essential micronutrient uptake and translocation and reducing As translocation. *Environ. Sci. Pollut. Res.* 25 26978–26991. 10.1007/s11356-018-2711-x 30008167

[B56] Nakano Y. Asada K. (1981). Hydrogen peroxide is scavenged by ascorbate-specific peroxidase in spinach chloroplasts. *Plant Cell Physiol.* 22 867–880. 10.1093/oxfordjournals.pcp.a076232

[B57] Nawaz F. Ashraf M. Y. Ahmad R. Waraich E. A. Shabbir R. N. Bukhari M. A. (2015). Supplemental selenium improves wheat grain yield and quality through alterations in biochemical processes under normal and water deficit conditions. *Food Chem.* 175 350–357. 10.1016/j.foodchem.2014.11.147 25577091

[B58] Nouet C. C. Motte P. Hanikenne M. (2011). Chloroplastic and mitochondrial metal homeostasis. *Trends Plant Sci.* 16 395–404. 10.1016/j.tplants.2011.03.005 21489854

[B59] Pastor V. Luna E. Mauch-Mani B. Ton J. Flors V. (2013). Primed plants do not forget. *Environ. Exp. Bot.* 94 46–56. 10.1016/j.envexpbot.2012.02.013

[B60] Pennanen A. Xue T. L. Hartikainen H. (2002). Protective role of selenium in plant subjected to severe UV irradiation stress. *J. Appl. Bot.* 76 66–76.

[B61] Pezzarossa B. Remorini D. Gentile M. L. Massai R. (2012). Effects of foliar and fruit addition of sodium selenate on selenium accumulation and fruit quality. *J. Sci. Food Agric.* 92 781–786. 10.1002/jsfa.4644 21953507

[B62] Pfaffl M. W. Horgan G. W. Dempfle L. (2002). Relative expression software tool (REST) for group-wise comparison and statistical analysis of relative expression results in real-time PCR. *Nucleic Acids Res.* 30:e36. 11972351 10.1093/nar/30.9.e36 PMC113859

[B63] Prochazkova D. Sairam R. K. Srivastava G. C. Singh D. V. (2001). Oxidative stress and antioxidant activity as the basis of senescence in maize leaves. *Plant Sci.* 161 765–771. 10.1016/S0168-9452(01)00462-9

[B64] Proietti P. Luigi N. Buono D. D. D’Amato R. Tedeschini E. Daniele D. B. (2013). Selenium protects olive (*Olea europaea* L.) from drought stress. *Sci. Hortic.* 164 165–171. 10.1016/j.scienta.2013.09.034

[B65] Rampino P. Pataleo S. Gerardi C. Mita G. Perrotta C. (2006). Drought stress response in wheat: physiological and molecular analysis of resistant and sensitive genotypes. *Plant Cell Environ.* 29 2143–2152. 10.1111/j.1365-3040.2006.01588.x 17081248

[B66] Rengasamy P. Olsson K. A. (1993). Irrigation and sodicity. *Aust. J. Soil Res.* 31 827–837. 10.1071/SR9930821

[B67] Saleh A. M. Madany M. M. Y. (2015). Coumarin pretreatment alleviates salinity stress in wheat seedlings. *Plant Physiol. Biochem.* 88 27–35. 10.1016/j.plaphy.2015.01.005 25634803

[B68] Shalaby T. Bayoumi Y. Alshaal T. Elhawat N. Sztrik A. El-Ramady H. (2017). Selenium fortification induces growth, antioxidant activity, yield and nutritional quality of lettuce in salt-affected soil using foliar and soil applications. *Plant Soil* 421 245–258. 10.1007/s11104-017-3458-8

[B69] Shekari F. Abbasi A. Mustafavi S. H. (2017). Effect of silicon and selenium on enzymatic changes and productivity of dill in saline condition. *J. Saudi Soc. Agric. Sci.* 16 367–374. 10.1016/j.jssas.2015.11.006

[B70] Shrivastava P. Kumar R. (2015). Soil salinity: a serious environmental issue and plant growth promoting bacteria as one of the tools for its alleviation. *Saudi J. Biol. Sci.* 22 123–131. 10.1016/j.sjbs.2014.12.001 25737642 PMC4336437

[B71] Sors T. G. Ellis D. R. Salt D. E. (2005). Selenium uptake, translocation, assimilation and metabolic fate in plants. *Photosynth. Res.* 86 373–389. 10.1007/s11120-005-5222-9 16307305

[B72] Sripinyowanich S. Klomsakul P. Boonburapong B. Bangyeekhun T. Asami T. Gu H. (2013). Exogenous ABA induces salt tolerance in indica rice (*Oryza sativa* L.): the role of OsP5CS1 and OsP5CR gene expression during salt stress. *Environ. Exp. Bot.* 86 94–105. 10.1016/j.envexpbot.2010.01.009

[B73] Subramanyam K. Arun M. Mariashibu T. S. Theboral J. Rajesh M. Singh N. K. (2012). Overexpression of tobacco osmotin (*Tbosm*) in soybean conferred resistance to salinity stress and fungal infections. *Planta* 236 1909–1925. 10.1007/s00425-012-1733-8 22936305

[B74] Subramanyam K. Sailaja K. V. Subramanyam K. Muralidhara Rao D. Lakshmidevi K. (2011). Ectopic expression of an osmotin gene leads to enhanced salt tolerance in transgenic chilli pepper (*Capsicum annum* L.). *Plant Cell Tissue Organ. Cult.* 105 181–192. 10.1007/s11240-010-9850-1

[B75] Szabados L. Savouré A. (2010). Proline: a multifunctional amino acid. *Trends Plant Sci.* 15 89–97. 10.1016/j.tplants.2009.11.009 20036181

[B76] Talame V. Ozturk N. Z. Bohnert H. Tuberosa R. (2007). Barley transcript profiles under dehydration shock and drought stress treatments: a comparative analysis. *J. Exp. Bot.* 58 229–240. 10.1093/jxb/erl163 17110587

[B77] Thitisaksakul M. Tananuwong K. Shoemaker C. F. Chun A. Tanadul O. U. Labavitch J. M. (2015). Effects of timing and severity of salinity stress on rice (*Oryza sativa* L.) yield, grain composition, and starch functionality. *J. Agric. Food Chem.* 63 2296–2304. 10.1021/jf503948p 25615402

[B78] Turakainen M. (2007). *Selenium and its Effects on Growth, Yield and Tuber Quality in Potato.* Ph. D. Dissertation, Department of Applied Biology, University of Helsinki Helsinki.

[B79] Ullrich W. R. (2002). “Salinity and nitrogen nutrition,” in *Salinity: Environment–Plants–Molecules* eds Läuchli A. Lüttge U. (Dordrecht: Kluwer) 229–248.

[B80] Valkama E. Kivimäenpää M. Hartikainen H. Wulff A. (2003). The combined effects of enhanced UV-B radiation and selenium on growth, chlorophyll fluorescence and ultrastructure in strawberry (*Fragaria × ananassa*) and barley (*Hordeum vulgare*) treated in the field. *Agric. For. Meteorol.* 120 267–278. 10.1016/j.agrformet.2003.08.021

[B81] Velikova V. Yordanov I. Edreva A. (2000). Oxidative stress and some antioxidant systems in acid rain-treated bean plants: protective role of exogenous polyamines. *Plant Sci.* 151 59–66. 10.1016/S0168-9452(99)00197-1

[B82] Wang C. Q. (2011). Water-stress mitigation by selenium in *Trifolium repens* L. *J. Plant Nutr. Soil Sci.* 174 276–282. 10.1002/jpln.200900011

[B83] Weatherley P. E. (1950). Studies in the water relations of the cotton plant. I. The field of measurement of water deficit in leaves. *New Phytol.* 49 81–97. 10.1111/j.1469-8137.1950.tb05146.x

[B84] Wojcik P. (2004). Uptake of mineral nutrients from foliar fertilization. *J. Fruit. Ornam. Plant Res.* 12 201–218.

[B85] Xue T. Hartikainen H. Piironen V. (2001). Antioxidative and growth promoting effect of selenium in senescing lettuce. *Plant Soil* 237 55–61. 10.1023/A:1013369804867

[B86] Yao X. Chu J. Wang G. (2009). Effects of selenium on wheat seedlings under drought stress. *Biol. Trace Elem. Res.* 130 283–290. 10.1007/s12011-009-8328-7 19214397

[B87] Zhang A. Jiang M. Zhang J. Tan M. Hu X. (2006). Mitogen-activated protein kinase is involved in abscisic acid-induced antioxidant defense and acts downstream of reactive oxygen species production in leaves of maize plants. *Plant Physiol.* 141 475–487. 10.1104/pp.105.075416 16531486 PMC1475456

[B88] Zhang H. X. Blumwald E. (2001). Transgenic salt-tolerant tomato plants accumulate salt in foliage but not in fruit. *Nat. Biotechnol.* 19 765–768. 10.1038/90824 11479571

[B89] Zhang H. X. Hodson J. N. Williams J. P. Blumwald E. (2001). Engineering salt-tolerant *Brassica* plants: characterization of yield and seed oil quality in transgenic plants with increased vacuolar sodium accumulation. *Proc. Natl. Acad. Sci. U.S.A.* 98 12832–12836. 10.1073/pnas.231476498 11606781 PMC60139

