# Supplementary figures and images for: Sodium Selenate Treatment Using a Combination of Seed Priming and Foliar Spray Alleviates Salinity Stress in Rice
Source: Front Plant Sci. 2019 Feb 11;10:116. doi: 10.3389/fpls.2019.00116 (PMC6378292; doi:10.3389/fpls.2019.00116)

## Supplementary file 1: Selection of optimal concentration of $\text{Na}_2\text{SeO}_4$

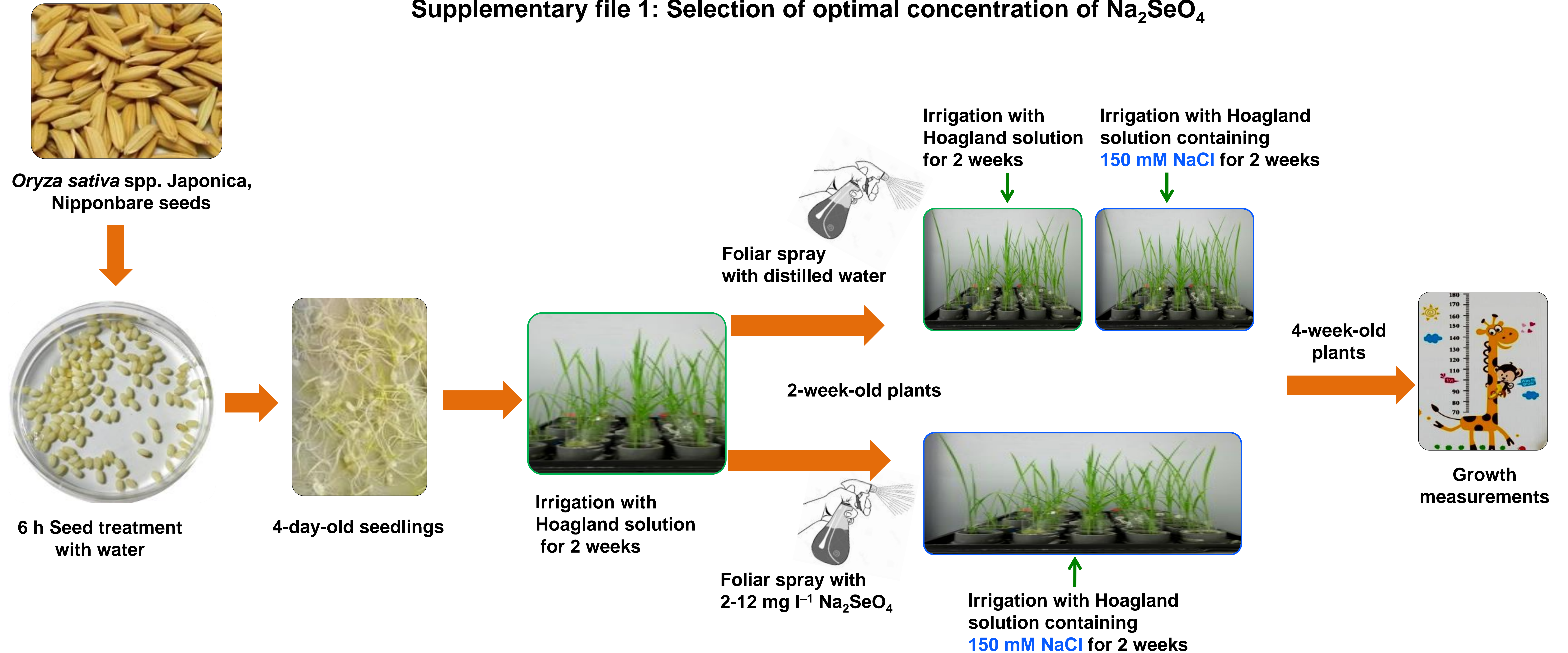

Supplement: FILE S1 — Selection of optimal concentration of sodium selenate (Na2SeO4). [file Data_Sheet_1.PDF]

## Supplementary file 2: Schematic representation of the experimental setup for control samples

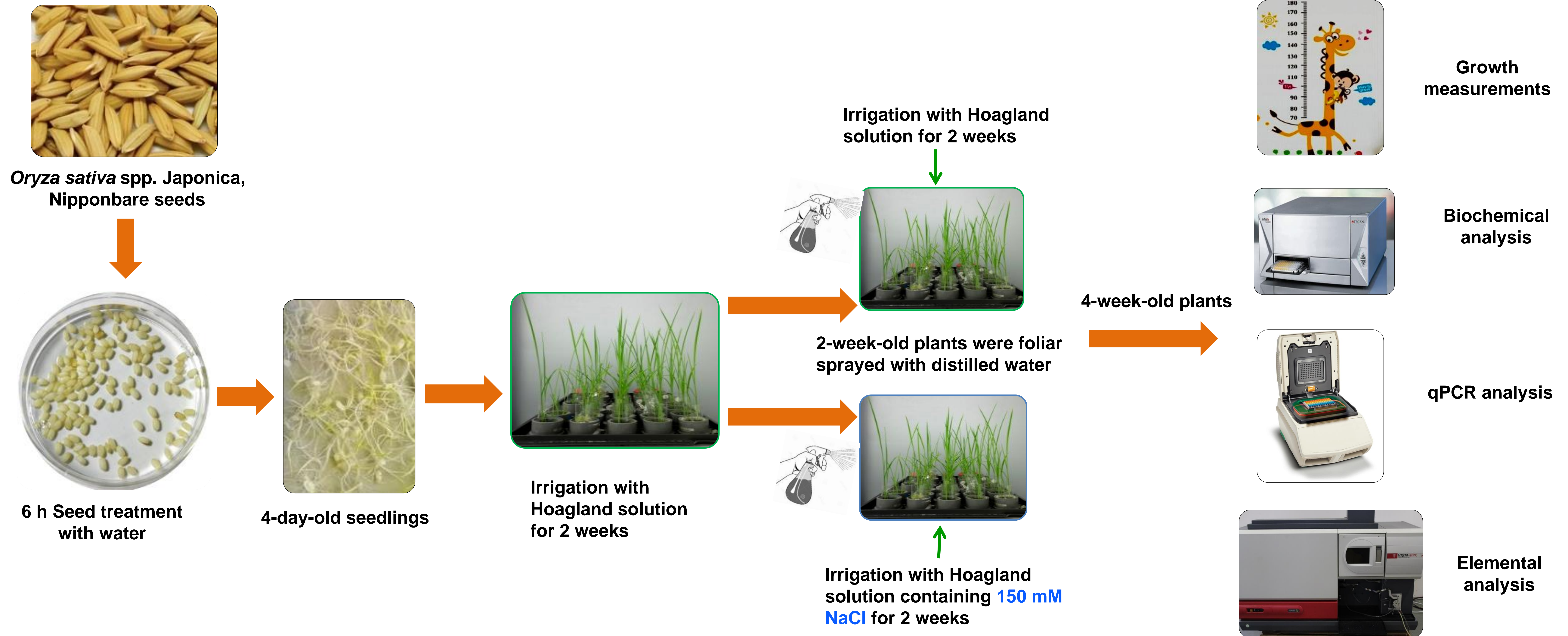

Supplement: FILE S2 — Schematic representation of the experimental setup for control samples. [file Data_Sheet_2.PDF]
