# Supplementary material for: Sodium Selenate Treatment Using a Combination of Seed Priming and Foliar Spray Alleviates Salinity Stress in Rice
Source: Front Plant Sci. 2019 Feb 11;10:116. doi: 10.3389/fpls.2019.00116 (PMC6378292; doi:10.3389/fpls.2019.00116)

Supplementary file 3: Schematic representation of Na_2SeO_4 application by mode I (foliar spray) to alleviate NaCl stress

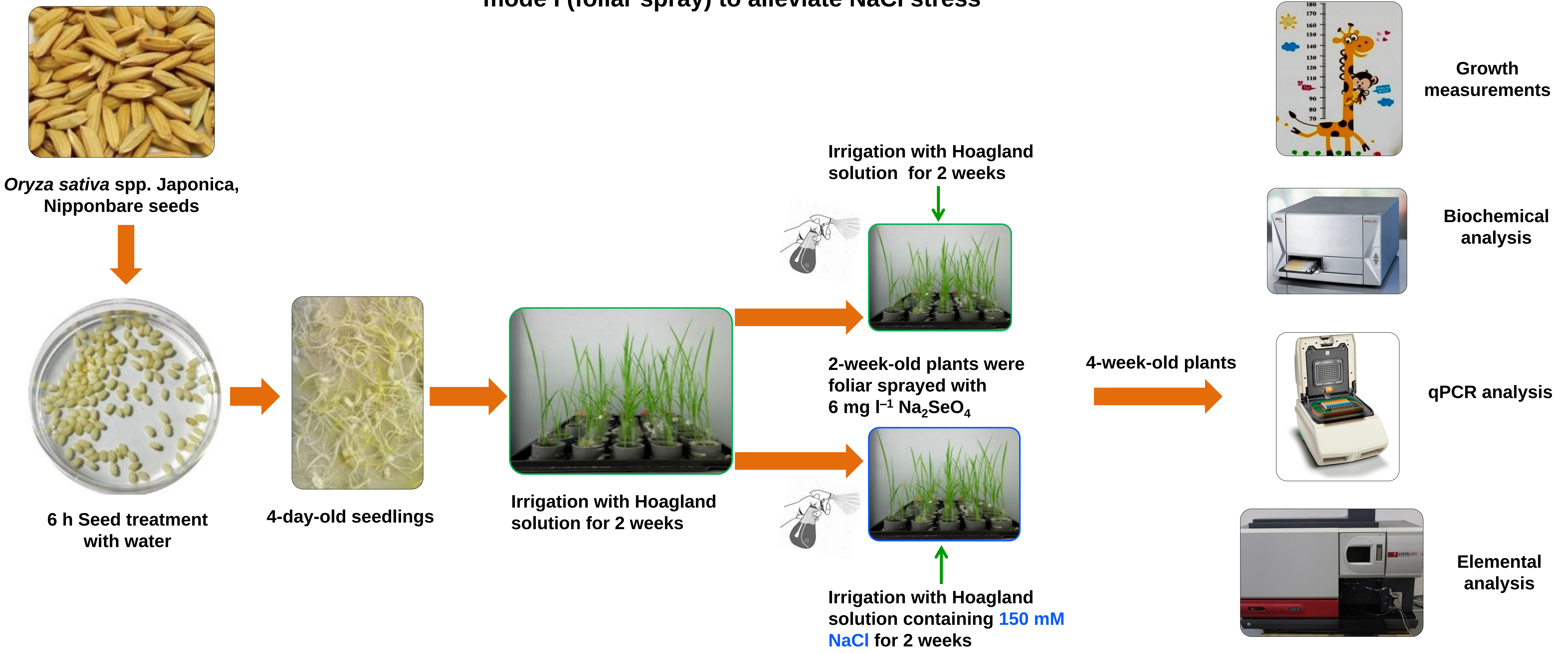

Supplement: FILE S3 — Schematic representation of Na2SeO4 application by mode I (foliar spray) to alleviate NaCl stress. [file Data_Sheet_3.PDF]
