# Supplementary material for: Sodium Selenate Treatment Using a Combination of Seed Priming and Foliar Spray Alleviates Salinity Stress in Rice
Source: Front Plant Sci. 2019 Feb 11;10:116. doi: 10.3389/fpls.2019.00116 (PMC6378292; doi:10.3389/fpls.2019.00116)
Supplement: FILE S5 — List of primers used for quantitative real time-PCR. [file Data_Sheet_5.PDF]

Supplementary file 5. List of primers used for quantitative real time-PCR.

Gene	Forward primer (5′ → 3′)	Reverse primer (5′ → 3′)
OsEXP (LOC_Os03g27010)	EVD910: TGTGAGCAGCTTCTCGTTTG	EVD911: TGTTGTTGCCTGTGAGATCG
OsEIF5C (LOC_Os11g21990.1)	EVD912: CACGTTACGGTGACACCTTTT	EVD913: GACGCTCTCCTTCTTCCTCAG
OsEXPNarsai (LOC_Os07g02340.1)	EVD914: AGGAACATGGAGAAGAACAAGG	EVD915: CAGAGGTGGTGCAGATGAAA
*OsNHX*1	P1132: CATTGATCAGGCTGCTGCTA	P1133: AGGAGAATGCAGGGACTTTG
